# Stage-specific regulation of signalling pathways to differentiate pluripotent stem cells to cardiomyocytes with ventricular lineage

**DOI:** 10.1186/s13287-022-02845-9

**Published:** 2022-05-06

**Authors:** Ramakanth Satthenapalli, Scott Lee, Jayanthi Bellae Papannarao, Timothy A. Hore, Akash Chakraborty, Peter P. Jones, Regis R. Lamberts, Rajesh Katare

**Affiliations:** 1 Department of Physiology, HeartOtago, School of Biomedical Sciences, University of Otago, 270, Great King Street, Dunedin, 9010 New Zealand; 2 Department of Anatomy, School of Biomedical Sciences, University of Otago, Dunedin, 9010 New Zealand; 3 Oklahoma Medical Research Foundation, Oklahoma City, USA

**Keywords:** Embryonic stem cells, Ventricular cardiomyocytes, Wnt signalling, Retinoic acid inhibition, Embryoid bodies

## Abstract

**Background:**

Pluripotent stem cells (PSCs) can be an ideal source of differentiation of cardiomyocytes in vitro and during transplantation to induce cardiac regeneration. However, differentiation of PSCs into a heterogeneous population is associated with an increased incidence of arrhythmia following transplantation. We aimed to design a protocol to drive PSCs to a ventricular lineage by regulating Wnt and retinoic acid (RA) signalling pathways.

**Methods:**

Mouse embryonic stem cells were cultured either in monolayers or three-dimensional hanging drop method to form embryonic bodies (EBs) and exposed to different treatments acting on Wnt and retinoic acid signalling. Samples were collected at different time points to analyse cardiomyocyte-specific markers by RT-PCR, flow cytometry and immunofluorescence.

**Results:**

Treatment of monolayer and EBs with Wnt and RA signalling pathways and ascorbic acid, as a cardiac programming enhancer, resulted in the formation of an immature non-contractile cardiac population that expressed many of the putative markers of cardiac differentiation. The population exhibited upregulation of ventricular specific markers while suppressing the expression of pro-atrial and pro-sinoatrial markers. Differentiation of EBs resulted in early foetal like non-contractile ventricular cardiomyocytes with an inherent propensity to contract when stimulated.

**Conclusion:**

Our results provide the first evidence of in vitro differentiation that mimics the embryonic morphogenesis towards ventricular specific cardiomyocytes through regulation of Wnt and RA signalling pathways.

**Supplementary Information:**

The online version contains supplementary material available at 10.1186/s13287-022-02845-9.

## Background

Cardiac regeneration via stem cell therapy is a promising field, and stem cell therapy using multipotent adult stem cells have been shown to ameliorate cardiac function. However, the efficacy of multipotent stem cells to differentiate into cardiovascular cells is still in debate, which is also reflected by the inconsistent results from the clinical trials [1–3]. Pluripotent stem cells (PSCs) are a potential source of cardiovascular cells due to their ability to differentiate into functional cardiovascular cells in vitro*,* and hence in replacing the loss of cardiomyocytes as well as vascular cells due to ischemia [4, 5]. However, one of the major challenges, and one that has received very little attention, is the need to generate chamber-specific cardiomyocytes. This is critical, as different chambers in the heart have different functions. For instance, the right atrium contains the pacemaker cells and many conduction cells required to initiate and conduct the electrical activity, respectively, whereas the ventricles are the heart's major pumping chambers containing the working cardiomyocytes. Unsurprisingly, our recent data show that the expression profiles of calcium handling proteins, crucial for contraction, are different between both chamber types in humans [6]. In general, PSCs yield a heterogeneous population of cardiomyocytes of which only 30–50% are ventricular cardiomyocytes [7, 8]. Since the majority of heart disease occurs in the ventricle, transplantation of a heterogeneous population with distinct functional and electrophysiological properties into the ventricle can contribute to cardiac arrhythmias [9, 10]. Recent animal studies have shown that exquisitely coordinated expression of various genes in a spatially and temporally controlled fashion is essential for normal cardiac development, with Wnt signalling, ascorbic acid (AA) and retinoic acid (RA) being key regulators of cardiogenesis [11, 12].

Wnt signalling has been reported to play a biphasic role in the differentiation of PSCs to cardiomyocytes where initial induction of Wnt signalling leads to the formation of mesoderm and the inhibition of Wnt signalling at later stages leads to differentiation to cardiomyocytes [13]. AA has been shown to enhance cardiac differentiation by upregulating the expression of transcription factors involved in the proliferation of cardiac progenitors in both mouse and human PSC models [14, 15]. On the other hand, RA has a significant role in specifying atrial and ventricular cardiomyocyte lineage in mouse and human PSCs. Positive signals of RA would lead to atrial specific cardiac differentiation, whereas inhibition of RA signalling drives the differentiation towards ventricular lineage [16]. These data therefore show that the individual modulation of Wnt, AA and RA signalling cascades can differentiate PSCs to cardiomyocytes. However, it is unknown whether combinatorial treatment with AA and temporal regulation of Wnt and RA signalling pathways, in a stage-specific manner, would lead to improved differentiation into a more homogeneous population of ventricular cardiomyocytes by suppressing the pacemaker population of cells. Therefore, the rationale of this study is to demonstrate if temporal regulation of Wnt and RA signalling pathway can differentiate PSCs to chamber-specific cardiac lineage.

In the current study, mouse embryonic stem cells (mESCs) were used as an in vitro PSC model. They were driven to cardiac lineage differentiation by regulating Wnt and RA signalling in the presence of AA as a cardiac programming enhancer. Cardiac differentiation in monolayer resulted in an immature non-contractile cardiac population that expressed all the putative markers of cardiac differentiation. They specifically exhibited upregulation of ventricular specific markers while suppressing the expression of pro-atrial and pro-sinoatrial markers.

## Methods

### mESC culture and characterization

A transgenic TNG-A (a clone of transgenic Nanog) mESC strain and its corresponding predecessor control E14 mESC strain were used for all the experiments [17]. The TNG-A strain displays a green fluorescence protein (GFP) reporter tagged to one allele of the *Nanog* gene, whereby GFP expression corresponds to the expression of pluripotent protein Nanog (Additional file 1: Figure S1) [18]. The loss of GFP indicates the corresponding loss of pluripotent Nanog expression. The mESCs were seeded and expanded in culture dishes that were pre-coated with autoclaved and sterile-filtered 0.1% gelatin (Sigma-Aldrich, NZ). The mESCs were supplemented with mESC medium [High glucose Dulbecco’s modified eagle’s medium (DMEM, Thermofisher, NZ), 15% fetal bovine serum, 1X non-essential amino acids (Sigma-Aldrich, NZ), 100 µM β-mercaptoethanol (Sigma-Aldrich, NZ), 1 μg/ml puromycin, and 10 ng/ml leukemia inhibitory factor (LIF) (Thermofisher, NZ)]. The mESCs were maintained at 37˚C in 5% CO_2_ in a humidified normoxic environment. The cells were dissociated with TrypLE (Thermofisher, NZ) and passaged once every 3–4 days when they attained 70% confluency to avoid spontaneous differentiation induced by over confluency. mESCs were characterized for their pluripotency before exposing them to differentiation conditions (Additional file 1: Figure S2).

### Cardiac differentiation of mESCs

#### Monolayer differentiation

**Fig. 1:**
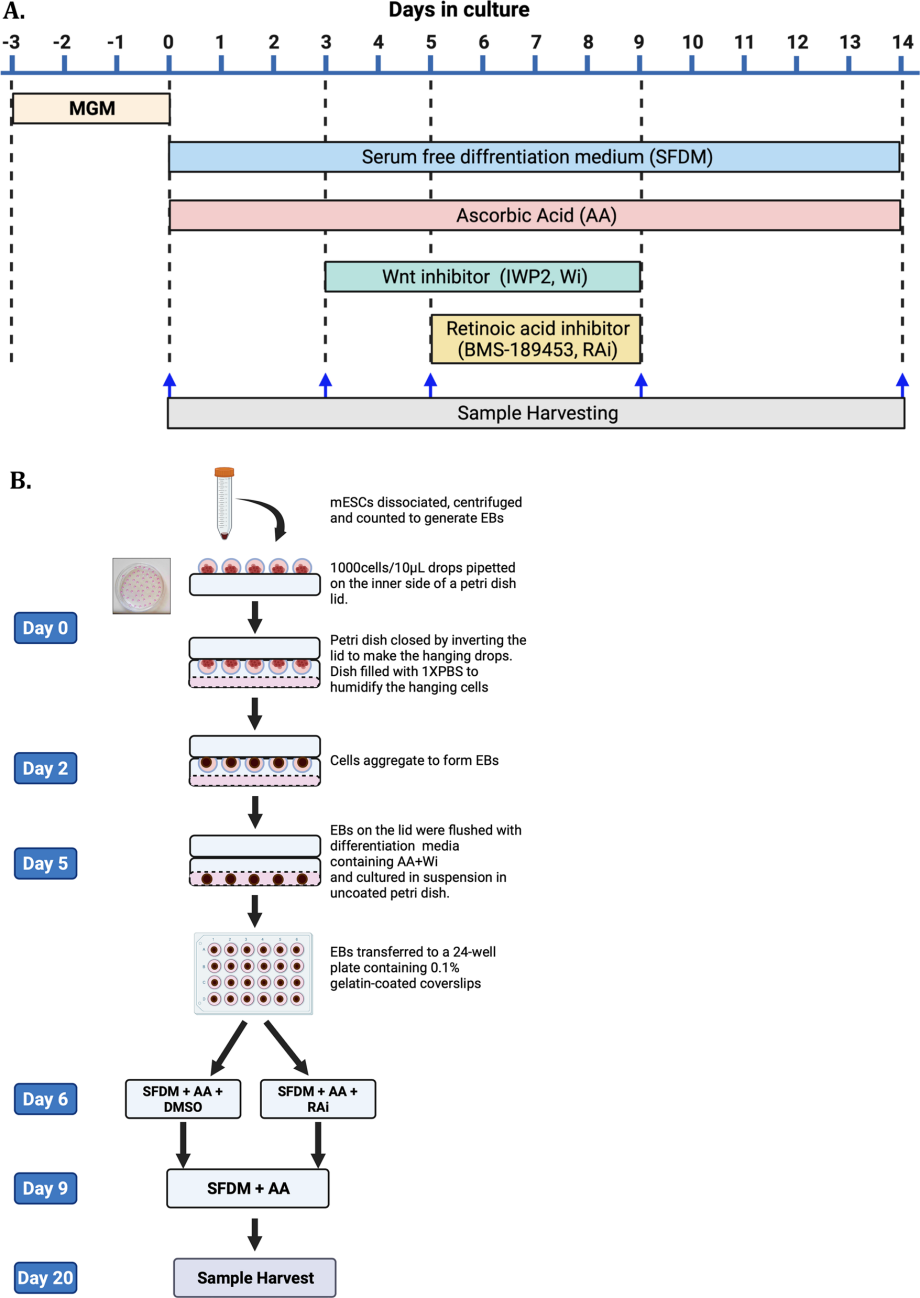
Schematic diagram of the protocols to differentiate mESCs to cardiovascular lineage. **A** Monolayer differentiation of mESCs to cardiomyocyte lineage. The mESCs were cultured in mESC growth medium (MGM) for three days (day − 3 to 0). On day 0, the medium was replaced with RPMI/B27-insulin as the serum-free differentiation medium (SFDM). Cells were treated with ascorbic acid (AA) from day 0, which continued until the end of the differentiation protocol (day 14). From days 3–5, cells were treated Wnt inhibitor (Wi), and from days 6–9, they were treated with retinoic acid inhibitor (RAi). **B** Three-dimensional differentiation of mESCs by EB method. EBs were generated by the hanging drop technique. On day 0, mESCs were dissociated, counted, and pipetted as drops of media on the inner side of a petri dish lid. The lid was inverted, and the petri dish was closed to form hanging drops. On day 2, spherical EBs was formed. On day 3, EBs were flushed with SFDM and cultured in suspension in an uncoated petri dish. On day 5, EBs were transferred to a 24-well plate containing 0.1% gelatin-coated coverslips. From day 5 to 30, EBs were cultured and differentiated in a monolayer. From day 6–9, cells were treated with RAi.

mESCs (200,000 cells/well) were seeded in a 24-well plate and allowed to grow for 3 days until they reached 80% confluent. To induce cardiac differentiation, the culture medium was replaced with serum-free medium, and cells were treated with AA (Sigma-Aldrich, NZ), IWP2 (Wnt inhibitor (Wi), Sigma-Aldrich, NZ) and BMS-189453 (RA inhibitor (RAi), Sigma-Aldrich, NZ) at different points as shown in Fig. 1A. Cells were harvested on days 3, 5, 9 and 14 for characterization.

#### Three-dimensional (3D) differentiation by embryoid body (EB) formation

EBs were generated using the three-dimensional hanging drop (HD) technique. For this, mESCs (1000 cells/10 µL) were suspended in the EB differentiation medium and placed as a drop (10 µL) on the inner side of the lid of a petri dish (Sigma-Aldrich, NZ). Around 40–50 drops were evenly placed per dish (Fig. 1B). The base of the petri dish was filled with 10 mL of sterile 1X phosphate buffered saline (PBS) as a source of humidity to the cell cluster in the hanging drops. The lid was then inverted, closed and cultured at 37 °C in a 5% CO_2_ incubator. Spherical EBs were visibly formed by day 2. The EBs on day 3 were gently flushed and plated on uncoated Petri dishes in EB differentiation medium supplemented with AA and Wi followed by treatment with RAi as shown in Fig. 1B.

### Quantitative real time-PCR (RT-PCR) analysis

Total RNA isolated using TRIzol (Thermofisher) was reverse-transcribed using PrimeScript™ RT Reagent Kit (Takara Bio, Japan). The resulting cDNA was amplified using SYBR green premix Ex Taq II (Takara Bio, Japan) in StepOne plus PCR machine (Thermo Fisher Scientific, USA) to quantify the expression of genes associated with pluripotency (*Nanog, Oct4 and Sox2*) and cardiac differentiation (Kinase Insert Domain Receptor (*KDR),* mesoderm posterior protein 1 (*Mesp-1),* islet-1 (*Isl-1*)*, Nkx2.5, T-Box Transcription Factor* 5 (*Tbx5*)*,* cardiac Troponin-T (*cTnT),* Myosin Regulatory Light Chain 2 Atrial isoform (*MLC-2a*)*,* Myosin regulatory Light Chain 2 Ventricular isoform (*MLC-2V*)*, and* Iroquois Homeobox 4 (*IRX4*). The day 0-mESCs was used as a negative control for the experiment to normalize the transcript levels of differentiated cells*.* The entire differentiation process, sample processing, and qPCR were independently repeated three times (*n* = 3). Data are presented as log fold change to day 0-mESCs. The list and sequence of all the primers used in this study are listed in Additional file 1: Table S1.

### Flow cytometry analysis

Cells in suspension were fixed with 4% PFA for 20 min at room temperature and probed with antibodies against pluripotency markers (Oct3/4, Sox2) and cardiac differentiation markers (T brachyury (T-Bry), KDR, ISL-1, Nkx2.5, cTnT, IRX4, MLC2V and connexin-43 (Cx43)). Details of antibodies, including the concentration used, are listed in Additional file 1: Table S2. To compensate for the use of multiple fluorescent antibodies, single stain controls comprising of antibody capture beads were used (AbC™ anti-mouse bead kit, Thermo Fischer Scientific, USA). Cell acquisition was performed using the flow cytometer (Gallios, Beckman Coulter Inc., USA). In total, 5000 to 50,000 events within the gate of interest were acquired depending on the sample type. Flow cytometer voltages were set using an unstained, single stained bead or secondary antibody controls so that the spill-over was minimum in the adjacent channels. Post-acquisition compensation and data analysis were performed using Flowjo 10.6.1 software (TreeStar, USA) [19].

### Immunofluorescence analysis

Monolayer cells seeded on glass coverslips in a 24-well plate were fixed with 4% PFA and permeabilized with pre-chilled methanol for intracellular staining. The cardiac cells of EBs were dissociated by collagenase IV/Trypsin treatment, singularized and seeded onto 0.1% gelatin-coated coverslips before fixation and permeabilization. After blocking with either 10% serum or 1%BSA, cells were incubated with primary antibodies against NkX2.5, Cx43, α-sarcomeric actin, IRX4 and MLC2V (all antibodies and concentrations listed in Additional file 1: Table S2) and incubated overnight at 4 °C. After washing, the cells were probed with secondary antibodies (1:500 dilution) conjugated with Alexa flour. Finally, cells were stained with DAPI (0.1 ug/mL, Thermo Fischer Scientific, USA) to visualize the nuclei and mounted on glass slides using Fluoromount G (Southern Biotech, USA). Images were captured in NikonA1R Resonant Scanner confocal microscope and analysed using ImageJ software, version 1.52 (NIH, USA). A control group for secondary antibodies (with the same labelling procedure without the primary antibody treatment, Additional file 1: Figure S3) was used to rule out the non-specific binding of the secondary antibody.

### Transmission electron microscopy (TEM)

The cells were fixed in 2.5% glutaraldehyde (primary fixative) and 1% osmium tetroxide (secondary fixative) dissolved in sodium cacodylate buffer. Post-fixation, the cells were dehydrated by washing with an ethanol series (50/70/90/100% ethanol, 5 min each). The cells were then treated with 100% propylene oxide, resin infiltrated, embedded in resin-filled silicone moulds, and finally were cured at 60 °C for 48 h. Post-curing, ultrathin sections (85 nm) were cut with an ultramicrotome (Leica EM UC7) and transferred to a copper grid (Sigma-Aldrich, NZ). The sections were then stained with 4% uranyl acetate for 25 min, rinsed in Milli-Q water, and transferred to 1% lead citrate for 5 min before rinsing in Milli-Q water and air drying. Images were captured with a Philip CM100 BioTWIN transmission electron microscope (Philips/FEI Corp., Eindhoven, Holland). The following parameters were measured; cell area (µm^2^), perimeter (µm), and circularity index [20]. The differentiated cells were identified based on the presence of multinucleated cardiac cells, increased cell size (area and perimeter), reduced circularity index, increased mitochondrial number, the presence of lipid droplets and glycogen bodies as some of the putative morphological characteristics observed during cardiac differentiation.

### Cytosolic Ca^2+^ imaging of differentiated cardiac cells

To measure the cytosolic Ca^2+^, differentiated cells were loaded with fluo-4-AM (2 μM, Thermofisher, NZ), a cell-permeable acetoxymethyl ester (AM) form of Ca^2+^ indicator for 10 min at room temperature. Cells were then washed twice with Krebs–Ringer HEPES (KRH) buffer to remove any de-esterified dye. Fluorescence of fluo-4 was detected by imaging the cells using CoolSNAP HQ2 CCD camera (Nikon). The analysis of cytosolic Ca^2+^ imaging was done using NIS-Elements AR Analysis 4.00.03 64-bit software (Coherent Scientific Pty. Ltd, Australia).

### Western blot analysis

mESCs at different time points of differentiation were homogenized in ice-cold RIPA lysis buffer supplemented with a 1% protease inhibitor cocktail (Sigma Aldrich, USA) [21]. The total protein was quantified by Bradford protein assay (BioRad, New Zealand). Twenty micrograms of protein were resolved by 15% SDS-PAGE and transferred to polyvinylidene fluoride membrane. After blocking and confirming the successful transfer of proteins using ponceau staining, membranes were probed with primary antibodies against CX43 (1:1000 dilution, Sigma Aldrich), MLC2V (1:1000 dilution, Abcam), and β-actin (1:1000 dilution, Santacruz Biotechnologies) overnight at 4 °C. Goat anti-rabbit secondary antibody (1:10,000 dilution, Abcam) was used for detection. The density of bands was analysed using ImageJ (NIH, United States) software and normalized to GAPDH.

### Experimental repeats and statistical analysis

The experimental repeats (*n*) were assay dependent. Quantitative real-time PCR, flow cytometry, and calcium imaging assays were performed with three independent repeats (*n* = 3). For TEM analysis, 10 cells in each sample were used for analysis. Spontaneous Ca^2+^ activity in the stimulated EBs was performed with at least 8 EBs. All the statistical analyses were done using Graph Pad Prism 9 software. Statistical significance was determined by (1) Student's *t*-test to identify the difference between two groups (2) a one-way ANOVA with Dunnett's or Sidak's multiple comparison post hoc test to identify the difference when there were more than two groups, and (3) a two-way ANOVA with Tukey's or Sidak's multiple comparison post hoc test to compare more than two groups with two independent variables. Data are presented as mean ± SEM. *P* < 0.05 was considered statistically significant.

## Results

### Monolayer differentiation of mESCs

#### Stage-specific expression of differentiated markers

**Fig. 2:**
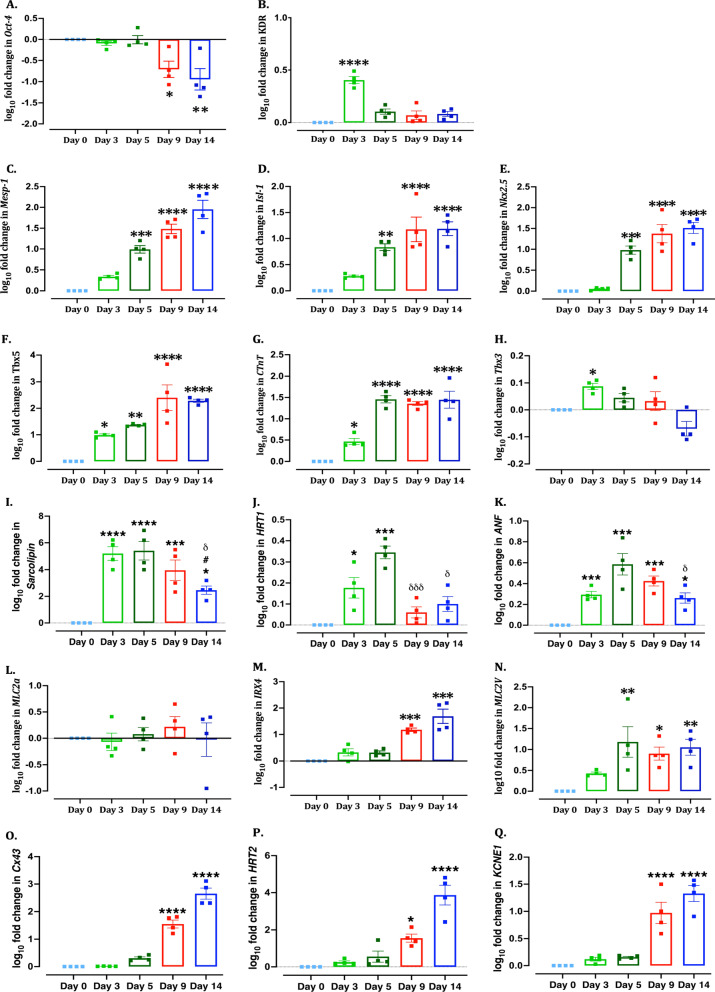
RT-PCR analysis of cardiac differentiation markers. Quantitative scatter plot bar graphs showing the expression of stage-specific cardiac differentiation genes on day 0, 3, 5, 9 and 14 following differentiation of mESCs in monolayers. The data were normalized to the gene expression of mESCs on day 0. Data were analysed by one-way ANOVA, Dunnett’s multiple comparisons test, *P ≤ 0.05, **P < 0.01, ***P < 0.001 and ****P < 0.0001 versus day 0 samples; δ < *P* < 0.05, δδδP < 0.001 versus day 3 samples; #*P* < 0.05 versus day 9 samples. *n* = 4 independent repeats

Serial monitoring of mESCs demonstrated the upregulation of putative cardiovascular markers in a stage-specific manner. qPCR analysis showed significant downregulation of the expression of pluripotent gene *Oct4* (Fig. 2A) and *Nanog* (Additional file 1: Figure S3) from day 9 of differentiation. AA treatment from day 0–3 (Fig. 1A) induced upregulation of cardiovascular precursor gene *KDR* (Fig. 2B). Wnt inhibition from day 3 (Fig. 1A) induced upregulation of cardiac mesoderm specific gene *Mesp-1* from day 5 (Fig. 2C). Further, Wnt inhibition induced upregulation of cardiac progenitor genes *Isl-1* (Fig. 2D) and *Nkx2.5* (Fig. 2E) and ventricular progenitor gene *Tbx5* (Fig. 2F), suggesting differentiation towards cardiac phenotype and, in particular, ventricular phenotype. The expression of both *Isl-1* and *Nkx2.5* remained upregulated until 14 days of differentiation (Fig. 2D&E), suggesting the cells still retain their progenitor phenotype. With the progression of differentiation, there was a significant upregulation of cardiac structural gene *cTnT* (Fig. 2G) as early as day 3, highlighting the importance of AA in initiating cardiac differentiation by increasing the number of CPC (also reported by Cao et al*.* [15]). While AA treatment-induced initial upregulation of pro-sinoatrial gene *Tbx3* (Fig. 2H) and atrial specific genes *Sarcolipin* (Fig. 2I), *HRT1* (Fig. 2J) and *ANF* (Fig. 2K), these were significantly downregulated following treatment with Wnt and RA inhibitors (Fig. 2H–K). Another atrial specific gene *MLC2a* showed no difference at any stage of differentiation (Fig. 2L). The ventricular specific differentiation of mESCs was further confirmed by significant upregulation of ventricular specific genes *IRX4* (Fig. 2M), *MLC2V* (Fig. 2N), Cx43 (Fig. 2O), HRT2 (Fig. 2P) and KCNE1 (Fig. 2Q). The upregulation of IRX4, Cx43, HRT2 and KCNE1 following treatment with RAi (Fig. 1A) highlights the inverse correlation between RA signalling and differentiation towards ventricular lineage and may indicate why the presence/absence of RA signalling would affect the atrial/ventricular cardiac population.

**Fig. 3:**
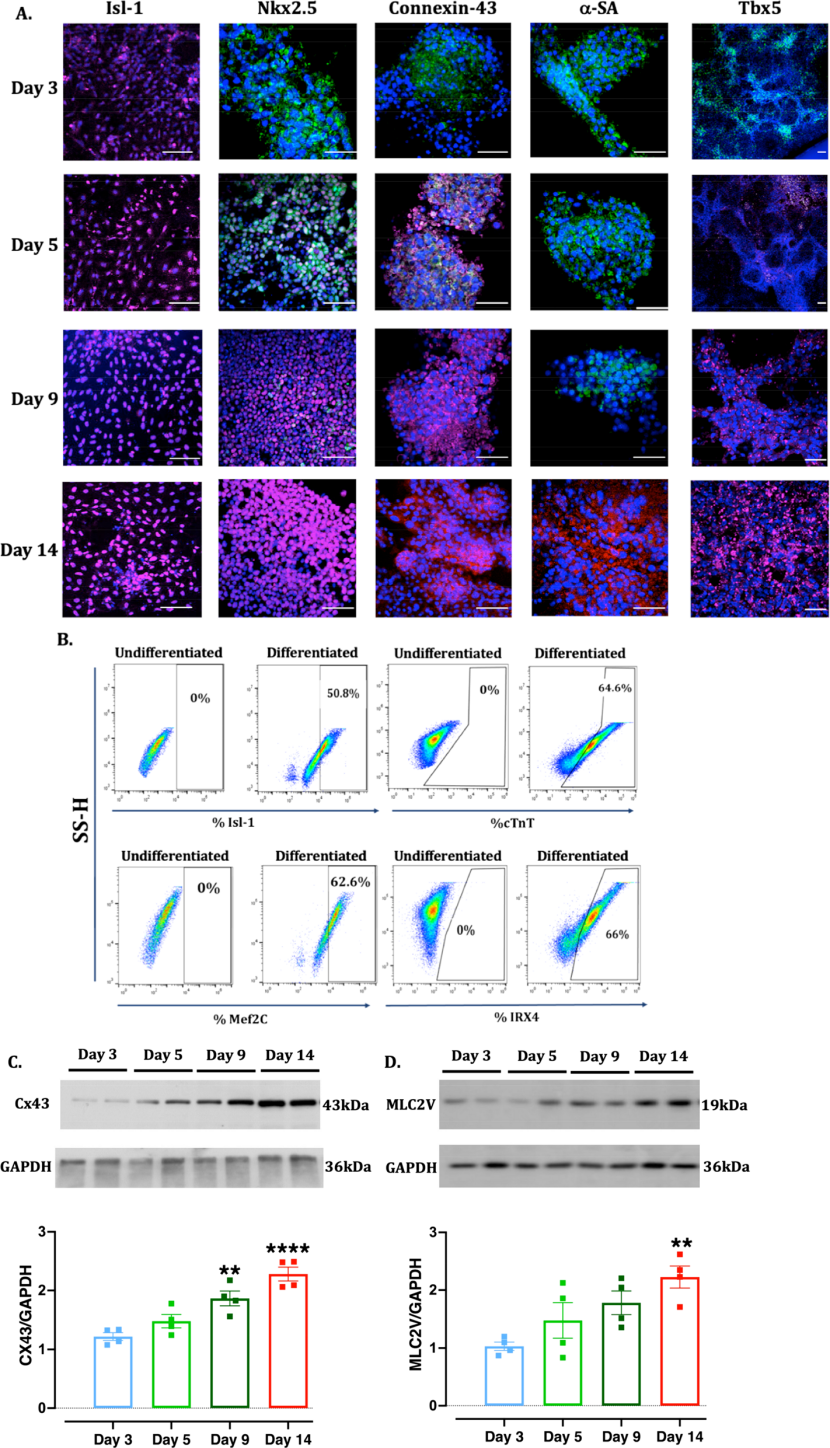
Expression of cardiomyocyte proteins. **A**, **B** Representative immunofluorescence and flow cytometry images showing the expression of cardiac transcription factors (Isl-1, Nkx2.5), cardiac structural protein (cardiac troponin T, cTnT), cardiac contractile protein (alpha-sarcomeric actin, α-SA) and left ventricular specific proteins (connexin-43, Tbx5 and IRX4) at on days 3, 5, 9 and 14 of differentiation. Secondary control was used as an experimental negative control in the absence of primary antibody to negate the non-specific binding of the secondary antibody and rule out false positive expression (Additional file 1: Figure S3). Nuclei were stained with DAPI. Initial green colour is due to the endogenous expression of GFP tagged pluripotent Nanog protein which gradually reduces and disappears with differentiation of cells. Positive staining is indicated by red colour. Scale bar = 50 µm. **C**, **D** Representative western blot images and quantitative scatter plot bar graphs showing the expression of ventricular specific proteins Cx43 (**C**) and MLC2V (**D**) at different time points

Importantly, observed changes in the gene expression was translated to proteins showing significant upregulation of cardiac transcription factors (Isl-1 (Fig. 3A, B) and Nkx2.5 (Fig. 3A)), structural protein (CTnT (Fig. 3B)), contractile protein ( α-sarcomeric actin (α-SA, Fig. 3A)) and ventricular specific proteins (connexin-43 (Fig. 3A, C and Additional file 1: Figure S8), Tbx5 (Fig. 3A), IRX4 (Fig. 3B), Mef2c (Fig. 3B) and MLC2V (Fig. 3D and Additional file 1: Figure S8) as observed by immunofluorescence at different time points (Fig. 3A) and on day 14 by flow cytometry (Fig. 3B) and western blot (Fig. 3C, D) analysis.

We further confirmed that mESCs do not "spontaneously" differentiate into cardiac lineage when cultured for 14 days in the absence of signalling regulators (Additional file 1: Figure S4).

#### The ultrastructure of differentiated mESCs revealed early-stage cardiomyocytes features

**Fig. 4:**
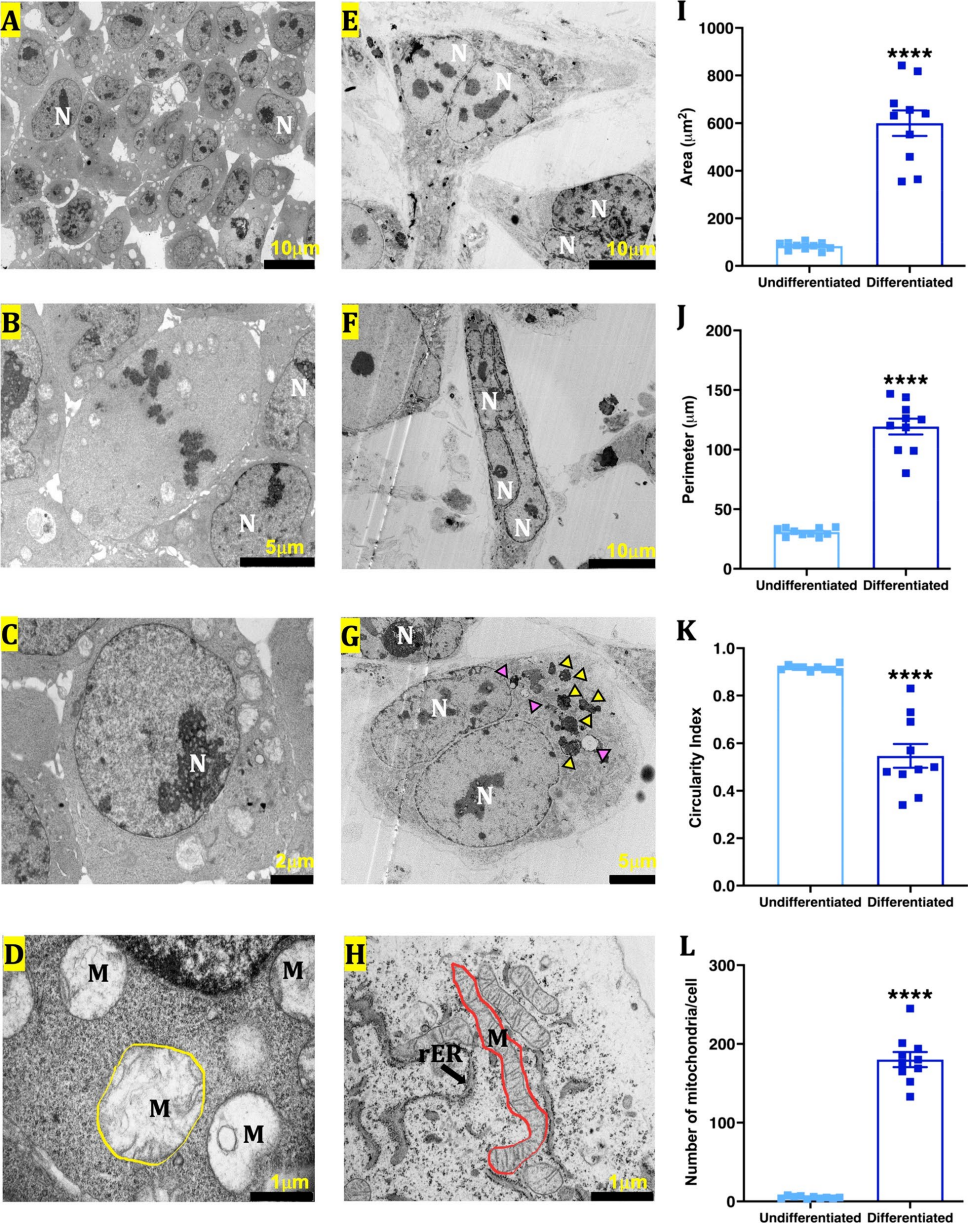
Ultrastructural features of mESCs differentiated in monolayers. **A–H—**representative transmission electron microscopy pictures of undifferentiated (**A–D**) and differentiated (**E–H**) mESCs. N—nucleus; M—mitochondria; ER—endoplasmic reticulum. **I–L**—quantitative scatter plot bar graphs showing cell area (**I**), perimeter (**J**), circularity index (**K**), and mitochondrial number (**L**). Data were analysed by unpaired *t*-test with Welch’s correction. Data presented as mean ± SEM. ****P < 0.0001 versus undifferentiated cells. *n* = 10 cells in each group

Differentiated mESCs (Fig. 4E–H) were larger compared to the small spherical undifferentiated mESCs (Fig. 4A–D) and displayed heterogeneous shapes (spherical, triangular or cylindrical) (Fig. 4E–H). Quantitative analysis showed an increase in cell area (83.18 ± 5.107 µm^2^ vs 600 ± 53.32 µm^2^, *P* < 0.0001) and perimeter (30.7 ± 1.075 µm vs 119.4 ± 6.596 µm, *P* < 0.0001), as well as decrease in circularity index (0.916 ± 0.004 vs 0.547 ± 0.05, *P* < 0.0001) (Fig. 4I–L). The majority of the differentiated cells were multinucleated, which is crucial before neonatal CMs are terminally differentiated. In contrast to the spherical immature mitochondria with little or no cristae in the undifferentiated mESCs (Fig. 4D), the mitochondria in the differentiated cells showed crisp membrane structures and cristae formation (Fig. 4H) and were high in number (Fig. 4L). The large number, size and mature cristae in the mitochondria of the differentiated cells could be attributed to their high demand for oxidative phosphorylation and hence, ATP production.

### Cardiac differentiation of mESCs from EBs

Monolayer differentiation did not show any maturation in morphology (Fig. 4) or Ca^2+^ activity (Additional file 1: Figure S5), which may be attributed to the monolayer cell culture not representing the in vivo embryonic niche. We next induced cardiac differentiation of mESCs using the EB method, which provides a 3D environmental niche. EB-differentiated mESCs exhibited mature morphology compared to the cells differentiated in monolayers **(**Additional file 1: Figure S6**)**. This was also confirmed by immunofluorescence analysis of cardiac transcription factors Nkx2.5 (Additional file 1: Figure S7A) and Isl-1 (Additional file 1: Figure S7B), and ventricular lineage markers IRX4 (Additional file 1: Figure S7C), MLC2V (Additional file 1: Figure S7C), Mef2c (Additional file 1: Figure S7D) and Cx43 (Additional file 1: Figure S7E). Flow cytometry analysis also confirmed the expression of IRX4 (Additional file 1: Figure S7F) and structural protein cTnT (Additional file 1: Figure S7F). Using this model, we determined the importance of RA inhibition (RAi) in ventricular specific differentiation of mESCs.

#### EB-differentiated mESCs displayed spontaneous contractility and cytosolic Ca^2+^ activity

**Fig. 5:**
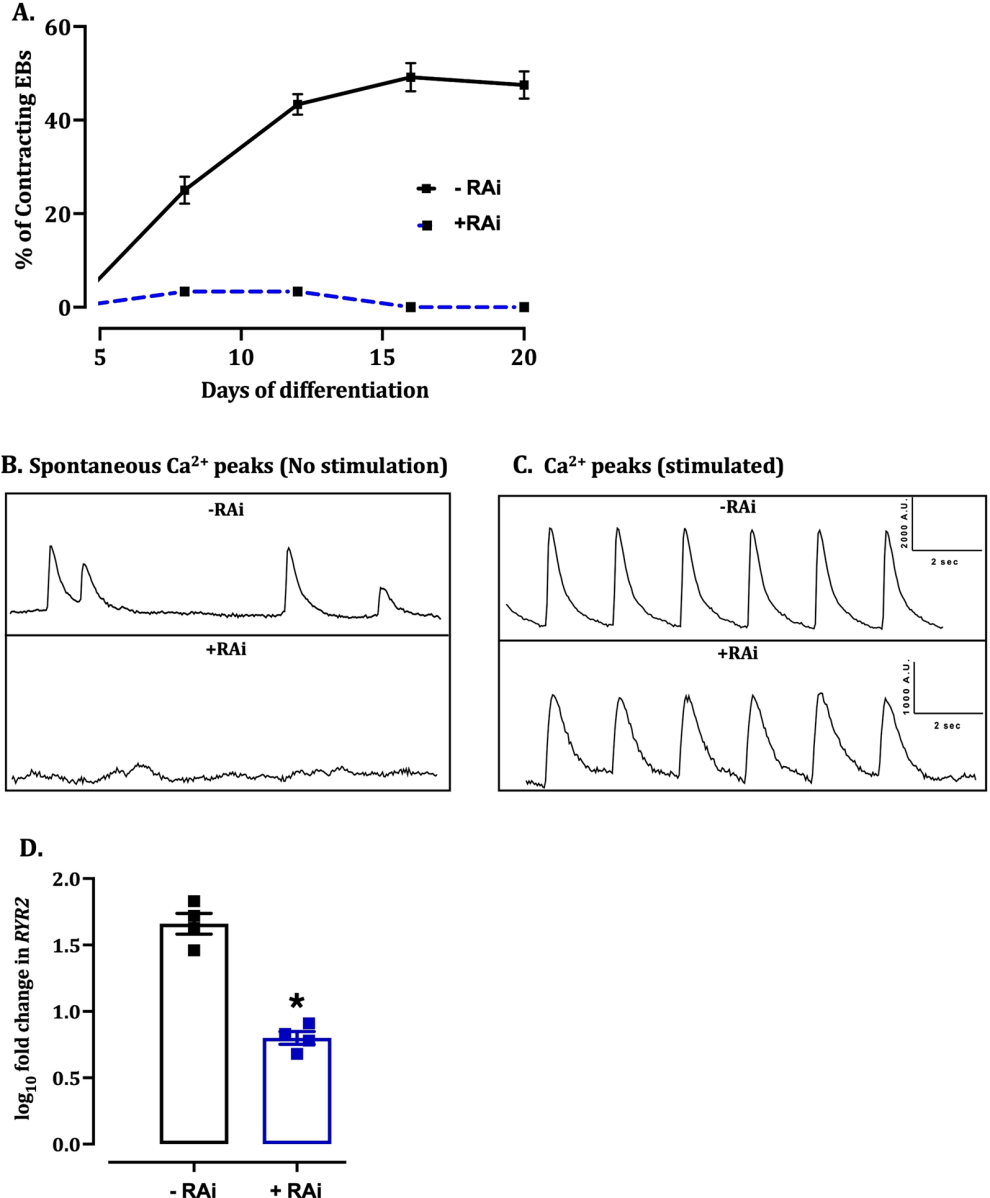
Characterization of mESCs differentiated by EB. **A** Quantitative line graphs showing the percentage of contracting EBs treated with RA inhibitor (+ RAi) or control (−RAi). Data were analysed by two-way ANOVA and presented as Mean ± SEM. *P < 0.05 versus previous time point; #*P* < 0.05 versus +RAi group at corresponding days. **B**, **C** Representative Ca^2+^ traces of a single EB dissected from −RAi and +RAi groups. The EBs were treated with Fluo-4 and electrically stimulated to visualize spontaneous Ca^2+^ release as functional characterization of contracting CMs. **D** Quantitative scatter plot bar graphs of RT-PCR analysis showing the expression of *RYR2* in the treatment groups. Data were analysed by unpaired *t*-test with Welch’s correction and presented as mean ± SEM. *P < 0.05 versus –Rai group. *n* = 4 independent repeats

In contrast to the cells differentiated in monolayers, EB-differentiated mESCs which were not treated with RA inhibitor (-RAi) showed spontaneous contractility, while this was not observed in the EB treated with RA inhibitor (+RAi, Fig. 5A). The area under the curve of averaged contracting EBs increased to 565 ± 25.2 in +RAi cells after 20 days of differentiation compared to 26.7 ± 5.8 −RAi cells (*P* < 0.001). This correlated with the spontaneous Ca^2+^ peaks in the −RAi (Fig. 5B) and higher expression of calcium release channel ryanodine receptor 2 (*RyR2*) (Fig. 5D). Spontaneous Ca^2+^ peaks were not observed in +RAi cells (Fig. 5B). However, when electrically stimulated, both −RAi and +RAi EBs demonstrated spontaneous contraction and hence the spontaneous Ca^2+^ peaks (Fig. 5C).

#### RAi downregulated sinoatrial and atrial specific gene/protein expression

**Fig. 6:**
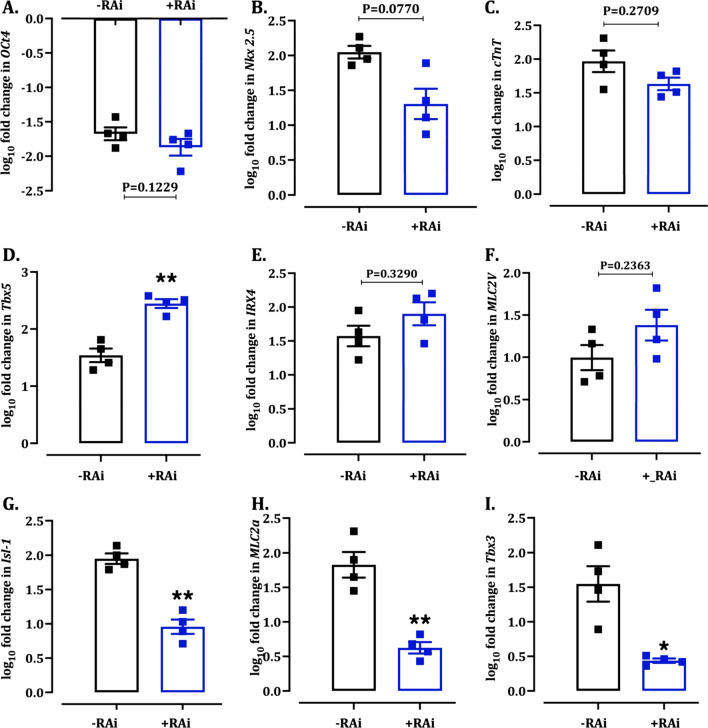
RT-PCR analysis of cardiac differentiation markers in mESCs differentiated by EB. Quantitative scatter plot bar graphs showing the expression of cardiac differentiation markers on day 14 of EB differentiation. The data were normalized to the gene expression of mESCs on day 0. Data were analysed by unpaired *t*-test and presented as mean ± SEM; *P < 0.05 and **P < 0.01 versus –Rai group. *n* = 4 independent repeats

Similar to the monolayer differentiation, RT-PCR analysis confirmed the downregulation of pluripotent gene *Oct4* (Fig. 6A) and upregulation of cardiac progenitor gene *Nkx2.5* (Fig. 6B) and structural gene *cTnT* (Fig. 6C) in both +RAi and −RAi groups. The +RAi group showed a significant upregulation in the expression of ventricular-specific progenitor gene *Tbx5* (Fig. 6D). At the same time, there was no significant difference in the expression of adult ventricular genes *IRX4* (Fig. 6E) and *MLC2V* (Fig. 6F) between the groups. However, RA inhibition prevented the upregulation of pro-atrial (*Isl-1* (Fig. 6G) and *MLC2a* (Fig. 6H)) and sinoatrial genes (T-box transcription factor 3 (*Tbx3*)) (Fig. 6I). This is supported by the absence of contracting EBs in +RAi EBs (Fig. 5A). These results suggest that AA and Wnt inhibition is sufficient to drive the differentiation towards ventricular phenotype. However, RA inhibition could act as a determining step to downregulate atrial and nodal subtypes in the differentiated cells, thus driving the cells towards ventricular specific differentiation.

#### RA inhibition increased mature cardiomyocytes phenotype of differentiated mESCs

**Fig. 7:**
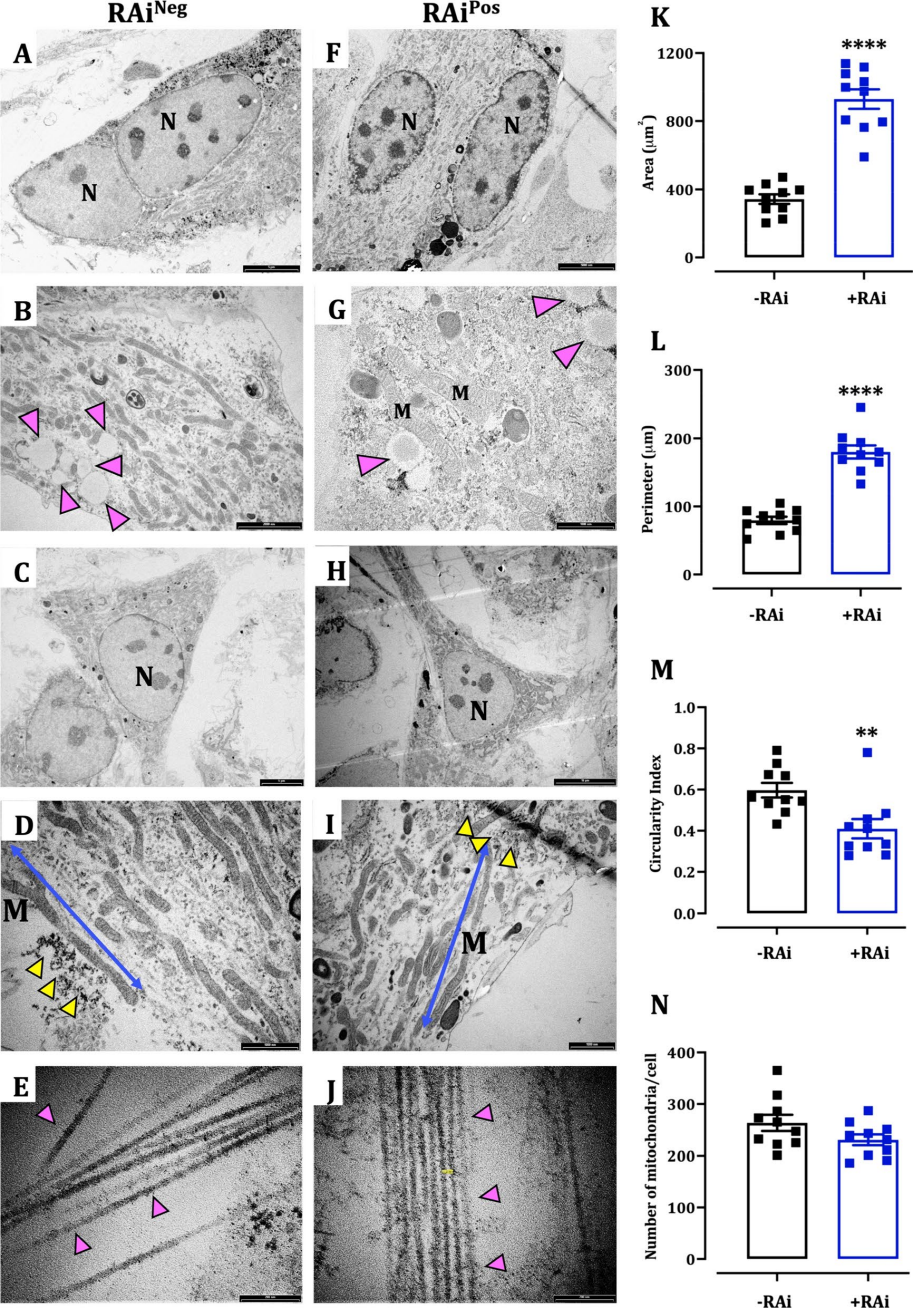
Ultrastructural features of mESCs differentiated by EB. **A–J—**representative transmission electron microscopy pictures of –Rai (**A–E**) and +RAi (**E–H**) cells. Yellow arrowhead—glycogen granules; pink arrowhead—collagen fibrils; N—nucleus; M—mitochondria; ER—endoplasmic reticulum. **K–N**—quantitative scatter plot bar graphs showing cell area (**K**), perimeter (**L**), circularity index (**M**) and mitochondrial number (**N**). Data were analysed by unpaired *t*-test with Welch’s correction and presented as mean ± SEM. **P < 0.01 and ****P < 0.0001 versus −RAi group. *n* = 10 cells in each group

Like monolayer-differentiated mESCs, the EB-differentiated mESCs were multinucleated with increased mitochondrial number and length, accumulation of lipid droplet, glycogen granule deposition, the SR calcium stores, and presence of clusters of rough-ER (Fig. 7A–J). Compared to −RAi cells, +RAi cells displayed a larger cell area (mean = 343 ± 28 µm^2^ vs mean = 929 ± 57 µm^2^, *P* < 0.0001, Fig. 7K) and perimeter (mean = 80 ± 5 µm vs mean = 180 ± 10 µm, *P* < 0.0001, Fig. 7L), and significantly smaller circularity index (mean = 0.6 ± 0.04 1 vs 0.41 ± 0.05, *P* = 0.006, Fig. 7M) suggesting a morphologically mature cardiomyocyte phenotype with RA inhibition. The mitochondrial number was not significantly different between both groups (Fig. 7N). Further, both the groups showed the presence of collagen fibrils following differentiation (Fig. 7E and J).

## Discussion

We have developed a unique combinatorial protocol to differentiate mESCs to cardiovascular lineage in a stage-specific manner. Although there are a plethora of protocols to differentiate PSCs to heterogeneous cardiomyocyte populations (nodal, atrial, and ventricular), to our knowledge, this is the first study to develop a protocol to differentiate PSCs to cardiomyocytes that are oriented towards ventricular-lineage without automaticity, via sequential modulation of the Wnt and RA signalling pathway in the presence of AA [15, 22, 23]. In this study, we differentiated the cells by growing them in monolayers and in EBs. In both models, RAi played a crucial role in differentiating the mESCs to a ventricular specific phenotype.

Using the monolayer cardiac differentiation protocol, we were able to demonstrate a stage-specific upregulation of cardiovascular precursors (*KDR* and *Mesp-1*), cardiac progenitors (*Isl-1, Nkx2.5,* and *Tbx5*), and ventricular cardiomyocyte markers (*Tbx5, IRX4,* and *MLC2V*) and downregulation of pluripotent markers (*Oct-4* and *Nanog*). Our results showed that AA specifies cardiovascular fate in mESCs, and inhibition of Wnt signalling induces cardiac mesoderm population. This is likely due to the interplay of epigenetic changes in DNA methylation of cardiac genes such as *Isl-*1 and the integrin signalling system [24]. In support of this, Isl-1 expression was upregulated at both gene and protein level as early as day 3 of differentiation in our study. Myocyte enhancer factor 2C (Mef2C) is a direct transcriptional target of Isl-1, and the expression of Mef2C is a sign of gradual cardiac looping morphogenesis during in vitro cardiac differentiation [25]. Sustained increase in Mef2C protein expression from day 3 to 14 of differentiation in our study signifies that the cardiac cells were undergoing an event of morphogenesis throughout the differentiation. *Nkx2-5* is one of the earliest known transcription factors required for cardiac cell specification and maintains the ventricle chamber identity. Nkx2.5 mutants had a reduced number of ventricular cardiomyocytes and excess atrial cells [26]. The double mutants for *Nkx2-5* and Heart and Neural Crest Derivatives Expressed 2 (*HAND2)* were shown to completely abolish the expression of the ventricular-specific homeobox gene *Irx4.* Nkx2.5 is also reported to be essential in maintaining the ventricle chamber identity [26, 27]. This evidence reiterates the significance of Nkx2.5 expression in early cardiac mesoderm specification, proliferation, heart tube morphogenesis, and ventricular chamber specification. In the current study, Nkx2.5 mRNA and protein levels were upregulated from day 5 of differentiation, implying the upregulation in the expression of a master regulator of various ventricle-specific genes. While Nkx2.5 specifies a ventricular chamber identity, Tbx5 and Tbx3 are the transcription factors involved in the development of the left ventricle [28, 29] and pacemaker cells [30], respectively. Upregulation of both Tbx5 and Tbx3 from day 3 differentiation suggested an additional role of AA in specifying the ventricular and pacemaker progenitor pool. While TBX5 gradually upregulated and sustained with RA inhibition, Tbx3 showed a gradual downregulation suggesting that the current treatment protocol does not promote specification or differentiation into pacemaker cells. This could partially explain the absence of automaticity in our differentiated cells. Indeed, EBs differentiated in the absence of RAi exhibited spontaneous contraction.

Further, RA inhibition also induced downregulation of pro-atrial gene *MLC2a* while upregulating pro-ventricular gene *MLC2V*. Although we did not explore the underlying mechanism by which RAi causes ventricular lineage differentiation, Wang et al*.* demonstrated that the formation of an inhibitory complex by IRX4 with retinoic X receptor (RXR) downregulated the expression of atrial-specific a unique myosin heavy chain (*AHMC1*), while promoting differentiation into the ventricular phenotype [31]. Based on this, upregulation of IRX4 in our study following RAi possibly increased its binding to RXR, inducing ventricular lineage differentiation. This is further supported by reduced *IRX4* and *MLC2V* in EB-differentiated cells in which RA was not inhibited.

Monolayer differentiation resulted in cells displaying the features of immature embryonic cardiomyocytes such as deposition of glycogen granules, lipid droplets, and rough ER. Cardiac glycogen is present at high levels during early to mid-gestation before falling to low levels at the time of birth, thereby playing a critical role as an energy source during cardiogenesis [32]. Lipid droplets act as a triglyceride pool for the mitochondria that may serve as a crucial source of signalling intermediates to govern the mitochondrial function and fatty acid oxidation by regulating the expression of peroxisome proliferator-activated receptor-gamma coactivator-1-alpha (PGC-1α) and PGC-1-beta(β) and the activity of Peroxisome proliferator-activated receptor-α (PPAR-α) [33]. Further, despite the expression of cTnT and α-sarcomeric actin, the ultrastructural analysis failed to identify sarcomeres. Asmuda et al. demonstrated the expression of cTnT in the nucleus of neonatal cardiomyocytes, indicating that cardiac troponin and tropomyosin could have an important cellular function(s) beyond Ca^2+^ regulation [34]. Although monolayer differentiated cardiomyocytes expressed RyR2 (Additional file 1: Figure S5C), a protein channel responsible for balancing Ca^2+^ homeostasis by effluxing excess of Ca^2+^ from its Ca^2+^ store sarcoplasmic reticulum (SR) upon stimulation [35], they failed to emit any Ca^2+^ peaks when stimulated with Ca^2+^ infusion. This could be possibly due to the RyR2 channels being not sensitive enough to respond to their external calcium cues. Interestingly, differentiation by EB formation showed mature cardiomyocyte phenotype, suggesting that mimicking embryonic morphogenesis results in the generation of morphologically and functionally mature CMs [36]. However, in spite of the expression of α-sarcomeric actin, these cells still lack sarcomeres, which is a functional unit of the mature cardiomyocytes. Therefore, future studies should aim to focus on molecular pathways that could trigger the formation of sarcomeres required for functional excitation–contraction coupling.

## Conclusions

The current study features a novel protocol to differentiate mESCs into cardiomyocytes of ventricular lineage. Our results indicate that stage-specific combinatorial treatment with AA and Wnt and RA inhibition can induce differentiation of mESCs to ventricular lineage-specific cardiomyocytes by suppressing the expression of pro-atrial and pro-sinoatrial lineage. Differentiation via monolayer generated non-contracting ventricular-specific cardiomyocytes. EB generated cardiomyocytes were spontaneously contracting, which was not observed when treated with RA inhibitor, thereby demonstrating the importance of RA inhibition in shifting differentiation of cardiomyocytes towards ventricular lineage. Moreover, the electrically stimulated +RAi cells showed spontaneous contraction and Ca^2+^ release similar to −RAiEBs. Next critical step is to determine if in vivo transplantation of the homogenous population of cardiomyocytes that specifically express ventricular lineage markers into the ischemic left ventricle shows superior fusion with the host cardiomyocytes and exhibit better therapeutic efficacy. This will allow for a significant advancement in increasing the efficacy of stem cells therapy in regenerating the diseased heart. Moreover, with the recent use of induced pluripotent stem cells (iPSCs) in regenerative therapy in the clinic and its ability to differentiate into cardiomyocytes, it will be crucial to test the efficacy of our differentiation strategy in iPSCs to determine its translation ability [37].

## Supplementary Information

**Additional file 1.** **Figure S1**. **A**. Representative bright-field image of TNG-A mESCs aggregated in spherical colonies. **B**. Acolony of TNG-A mESCs showing the endogenous expression of pluripotent nanog protein due to the ***GFP*** transgene tagged to *nanog*. Scale bar=100μm. **Figure S2**. **A&C**. Representative immunofluorescence images of mESCs on day 0 (**A**) and day 14 (time control, **C**) showing the expression of pluripotent markers (Oct4 and Sox2). Specificity of the antibody was confirmed by staining a independent samples with secondary controls only. B&D. Quantitative scater plot bar graphs of RT-PCr analysis showing the expression of pluripotent markers gene *Nanog, Oct-4* and *Sox-2* in of mESCs on day 0 (B) and day 14 (time control, **G**). Mature mouse cardiomyocyte cell line HL-1 cells were used as negative controls. **Figure S3**. Representative confocal microscopy images showing the cells at different days of differentiation that are stained with secondary antibodies with the same labelling procedure without the primary antibody treatment, to rule out the non-specific binding of the secondary antibody. **Figure S4**. Quantitative scatter plot bar graphs of RT-PCR analysis showing the expression of cardiac differentiation genes in mESCs grown in differentiation medium with treatments (differentiated) and mESCs grown without any treatment (time control). Data were analyzed by unpaired t-test presented as Mean ± SEM. *P<0.05. n=4 independent repeats. **Figure S5**. **A**. Representative image of Ca^2+^ activity in a single differentiated mESC-CM on day 14 (**A**). The cells did not show any propensity to spontaneously release the calcium when induced with different concentrations of Ca^2+^. **B**. Quantitative scatter plot bar graphs showing a significant response of differentiated cells to caffeine-induced Ca^2+^ release **C**. Quantitative scatter plot bar graphs of RT-PCR analysis showing the expression of *RYR2* after 14 days of differentiation. Data were analyzed by unpaired t-test with Welch’s correction. **P<0.01 and ****P<0.0001 vs. differentiated cells. Data presented as Mean ± SEM. n=35 cells from three independent experiments in B n=4 independent experiments in **C**. **Figure S6**. Representative bright field microscopic images of m-ESC over 14days of cultivation by EB method. On day 2 (**A**) the EBs were generated and transferred to a petri dish. The colony was preserved and the EB was still in three-dimensional shape on day 3 (**B**), On day 4 (**C**) the EBs were transferred to gelatin coated plates and finally on day 5(**D**) the EB adhering to the plate was observed. From day 6 (**E**) the images were taken at a magnification of 40x to observe closer morphological changes. Cells looked more spherical and the cells were observed very close together. Over the period of day 7(**F**), 8 (**G**), 9(**H**), 10(**I**), 11(**J**), 12(**K**), 13(**L**), 14(**M**), the morphology of cells became more elongated and also the myotube formation was observed. Clear morphological differences can be seen comparing undifferentiated ESC (**N**) and differentiated cardiomyocytes on day14 (**O**). Scale bars are 500 μM for A-D, 200 μM for E-M and 00 μM for N&O. **Figure S7**. **A–B**. Representative confocal microscopy images showing the expression of cardiac transcription factors Nkx2.5 (**A**) and Isl-1 (**B**) in differentiated and undifferentiated mESCs by EB method. **C**. Representative confocal microscopy images showing the expression of ventricular cardiomyocytes markers IRX4 and MLC2V following differentiation of mESCs by EB method in both +RAi and -RAi groups. **D–E**. Representative confocal microscopy images showing the expression of ventricular cardiomyocytes markers Mef2c (**D**) and Cx43(**E**) in differentiated and undifferentiated mESCs by EB method. **F**. Representative flowcytometry gating images showing the expression of left ventricular cardiomyocytes marker IRX4 and cTnt following differentiation of mESCs by EB method in both +RAi and -RAi groups. **Figure S8**. Uncropped western blots of Connexin43 (Cx43) and GAPDH represented in Figure 3C (**A**) and MLC2V and GAPDH represented in Figure 3D (**B**).

## Data Availability

All the data are included in the manuscript.

## Abbreviations

AA: Ascorbic acid
AHMC1: Atrial-specific a unique myosin heavy chain
AM: Acetoxymethyl ester
cTnT: Cardiac troponin-T
Cx43: Connexin-43
DMEM: Dulbecco’s modified eagle’s medium
EB: Embryonic bodies
GFP: Green fluorescence protein
HAND2: Heart and neural crest derivatives expressed 2
HD: Hanging drop
IRX4: Iroquois homeobox 4
Isl-1: Islet-1
KDR: Kinase insert domain receptor
KRH buffer: Krebs-Ringer HEPES buffer
Mef2C: Myocyte enhancer factor 2C
Mesp-1: Mesoderm posterior protein 1
MLC-2a: Myosin regulatory light chain 2 atrial isoform
MLC-2V: Myosin regulatory light chain 2 ventricular isoform
PBS: Phosphate buffered saline
PGC-1α: Peroxisome proliferator-activated receptor-gamma coactivator-1-alpha
PGC-1β: Peroxisome proliferator-activated receptor-gamma coactivator-1-beta
PPAR-α: Peroxisome proliferator-activated receptor-α
RA: Retinoic acid
Rai: RA inhibitor
RXR: Retinoic X receptor
RYR2: Ryanodine receptor
α-SA: α-Sarcomeric actin
SR: Sarcoplasmic reticulum
T-Bry: T brachyury
Tbx3: T-box transcription factor 3
Tbx-5: T-box transcription factor 5
TNG-A: A clone of transgenic Nanog
