# Supplementary material for: Stage-specific regulation of signalling pathways to differentiate pluripotent stem cells to cardiomyocytes with ventricular lineage
Source: Stem Cell Res Ther. 2022 May 6;13:185. doi: 10.1186/s13287-022-02845-9 (PMC9077927; doi:10.1186/s13287-022-02845-9)

Stage-specific regulation of signalling pathways to differentiate pluripotent stem cells to cardiomyocytes with ventricular lineage

Ramakanth Satthenapalli[1], Scott Lee[1], Jayanthi Bellae Papannarao[1], Tim Hore[2], Akash Chakraborty[1], Peter P. Jones[1], Regis R. Lamberts[1], Rajesh Katare[1]

Department of Physiology[1], HeartOtago and Department of Anatomy[2], School of Biomedical Sciences, University of Otago, Dunedin, New Zealand 9010

Corresponding author:

Rajesh Katare

Department of Physiology-HeartOtago

270, Great King Street, Dunedin, New Zealand 9010

Tel - +64-3-4797292

Email – rajesh.katare@otago.ac.nz

Supplemental Table 1 – Sequence of the primers used in the study

Oligo Name	Sequence
Nanog	F- CCAGAACCAGAGAATGAAATC<br>R- TGGTGGTAGGAAGAGTAAAG
Oct4	F- GATCACCCTGGGATATACAC<br>R- GCTTTGCATATCTCCTGAAG
Sox2	F- ATAATAACAATCATCGGCGG<br>R- AAAAAGAGAGAGGCAAACTG
T-Bry	F- TGGGAGTGGAGAGTTTAGCA<br>R- AATGTTTGCACCTCCATCAA
KDR	F- AATGGTACAGAAATGGAAGG<br>r- GCATCTCTTTCAGTCACTTC
Mesp-1	F- GTACACGCTCTAAAGATGAAG<br>R- TTTTGACACTAGCACAATCG
Isl1	F- CAGCAACTGGTCAATTTTTC<br>R- TGAATGAATGTTCCTCATGC
Nkx2.5	F- CACTTTATTGACGTAGCCTG<br>R- AAAACATAAATACGGGTGGG
cTnt	F- AGAGTATTCACAACCTGGAG<br>R- GAGTTTTGGAGACTTTCTGG
MLC2a	F- TCTGGAATTCAAGGAAGCCT<br>R- CTTCCTCCGGAAACACTTACC
Tbx3	F- AGACACAAAAAGGAGAATGG<br>R- AATCTTTGAGGTTCGATGTC
Tbx5	F- CCAGAATCACAAGATCACAC

	R- CGATACTCTTTACTTTGCATCC
IRX4	F- CATCTGCTTTCTACTCTCTG<br><br>R- TACCTGTCATATGGGTACTG
MLC2V	F- AATTAACTTCACCGTGTTCC<br><br>R- GTTGAGAATGGTCTCTTCAG
RyR2	F- CGTTGCGTATCTTAGCTATTC<br><br>R- GGACTTTCAAGCAGTAGTATC
18S rRNA	F- CAGTTATGGTTCCTTTGGTC<br><br>R- TTATCTAGAGTCACCAAGCC
CD31	F-CATCGCCACCTTAATAGTTG<br><br>R- CCAGAAACATCATCATAACCG
Calponin-1	F- TGCTGAAGTAAAGAACAAGC<br><br>R- CATTGACCTTCTTCACAGAAC

Supplementary Table 2 – Detail of the antibodies used in the study

S.No.	Type of antibody	Name	Species raised in	Dilution	Manufacturer and catalogue number
1	Unconjugated	Oct3/4	Mouse	1:100	SANTA CRUZ BIOTECHNOLOGY, INC (sc-5279)
2	Unconjugated	Sox2	Rabbit	1:100	ThermoFisher scientific (Catalo # 48-1400)
3	Conjugated	T-Bry-PE	Mouse	1:10	R&D SYSTEMS (IC0041P)
4	Conjugated	KDR	Mouse	1:100	eBiosciences (17-5821-81)
5	Unconjugated	Isl-1	Rabbit	1:100	Abcam (ab109517)
6	Unconjugated	Nkx2.5	Mouse	1:100	Abcam (ab91196)
7	Unconjugated	Nkx2.5	Rabbit	1:100	Abcam (ab97355)
8	Conjugated	cTnT	Mouse	1:100	Miltenyi Biotech (130-106-687)
9	Unconjugated	IRX4	Rabbit	1:100	Abcam (ab123542)
10	Unconjugated	MLC2V	Rabbit	1:100	Abcam (ab79935)
11	Unconjugated	Connexin 43	Rabbit	1:100	Sigma-Aldrich (SAB4501175)
12	Conjugated	CD34-FITC	Mouse	1:100	eBiosciences (Catalogue # 11-0341-82)
13	Conjugated	CD31-PE-Cy7	Mouse	1:100	eBiosciences (Catalogue # 25-0311-82)
14	Unconjugated	α-SMA	Mouse	1:100	Sigma-Aldrich (A52280)

Supplementary Figure Legends

Supplementary Figure 1.

A. Representative bright-field image of TNG-A mESCs aggregated in spherical colonies. **B.** A colony ofTNG-A mESCs showing the endogenous expression of pluripotent nanog protein due to the *GFP* transgene tagged to *nanog*. Scale bar=100µm.

Supplementary Figure 2.

A&C. Representative immunofluorescence images of mESCs on day 0 (**A**) and day 14 (time control, **C**) showing the expression of pluripotent markers (Oct4 and Sox2). Specificity of the antibody was confirmed by staining a independent samples with secondary controls only. B&D. Quantitative scater plot bar graphs of RT-PCr analysis showing the expression of pluripotent markers gene *Nanog*, *Oct-4* and *Sox-2* in of mESCs on day 0 (**B**) and day 14 (time control, **G**). Mature mouse cardiomyocyte cell line HL-1 cells were used as negative controls.

Supplementary Figure 3.

Representative confocal microscopy images showing the cells at different days of differentiation that are stained with secondary antibodies with the same labelling procedure without the primary antibody treatment, to rule out the non-specific binding of the secondary antibody.

Supplementary Figure 4.

Quantitative scatter plot bar graphs of RT-PCR analysis showing the expression of cardiac differentiation genes in mESCs grown in differentiation medium with treatments (differentiated) and mESCs grown without any treatment (time control). Data were analyzed by unpaired t-test presented as Mean ± SEM. *P<0.05. n=4 independent repeats.

Supplemental Figure 5.

A. Representative image of Ca^{2+} activity in a single differentiated mESC-CM on day 14 (**A**). The cells did not show any propensity to spontaneously release the calcium when induced with different concentrations of Ca^{2+}. **B.** Quantitative scatter plot bar graphs showing a significant response of differentiated cells to caffeine-induced Ca^{2+} release **C.** Quantitative scatter plot bar graphs of RT-PCR analysis showing the expression of *RYR2* after 14 days of differentiation. Data were analyzed by unpaired t-test with Welch's correction. **P<0.01 and ****P<0.0001

vs. differentiated cells. Data presented as Mean ± SEM. n=35 cells from three independent experiments in **B** n=4 independent experiments in **C**.

Supplementary Figure 6.

Representative bright field microscopic images of m-ESC over 14days of cultivation by EB method. On day 2 (**A**) the EBs were generated and transferred to a petri dish. The colony was preserved and the EB was still in three-dimensional shape on day 3 (**B**), On day 4 (**C**) the EBs were transferred to gelatin coated plates and finally on day 5(**D**) the EB adhering to the plate was observed. From day 6 (**E**) the images were taken at a magnification of 40x to observe closer morphological changes. Cells looked more spherical and the cells were observed very close together. Over the period of day 7(**F**), 8 (**G**), 9(**H**), 10(**I**), 11(**J**), 12(**K**), 13(**L**), 14(**M**), the morphology of cells became more elongated and also the myotube formation was observed. Clear morphological differences can be seen comparing undifferentiated ESC (**N**) and differentiated cardiomyocytes on day14 (**O**). Scale bars are 500 µM for A-D, 200µM for E-M and 00µM for N&O.

Supplemental Figure 7.

A-B. Representative confocal microscopy images showing the expression of cardiac transcription factors Nkx2.5 (**A**) and Isl-1 (**B**) in differentiated and undifferentiated mESCs by EB method. **C.** Representative confocal microscopy images showing the expression of ventricular cardiomyocytes markers IRX4 and MLC2V following differentiation of mESCs by EB method in both +RAi and -RAi groups. **D-E.** Representative confocal microscopy images showing the expression of ventricular cardiomyocytes markers Mef2c (**D**) and Cx43(**E**) in differentiated and undifferentiated mESCs by EB method. **F.** Representative flowcytometry gating images showing the expression of left ventricular cardiomyocytes marker IRX4 and cTnt following differentiation of mESCs by EB method in both +RAi and -RAi groups.

Supplemental Figure 8

Uncropped western blots of Connexin43 (Cx43) and GAPDH represented in Figure 3C (**A**) and MLC2V and GAPDH represented in Figure 3D (**B**).

Supplementary Figure 1

A.

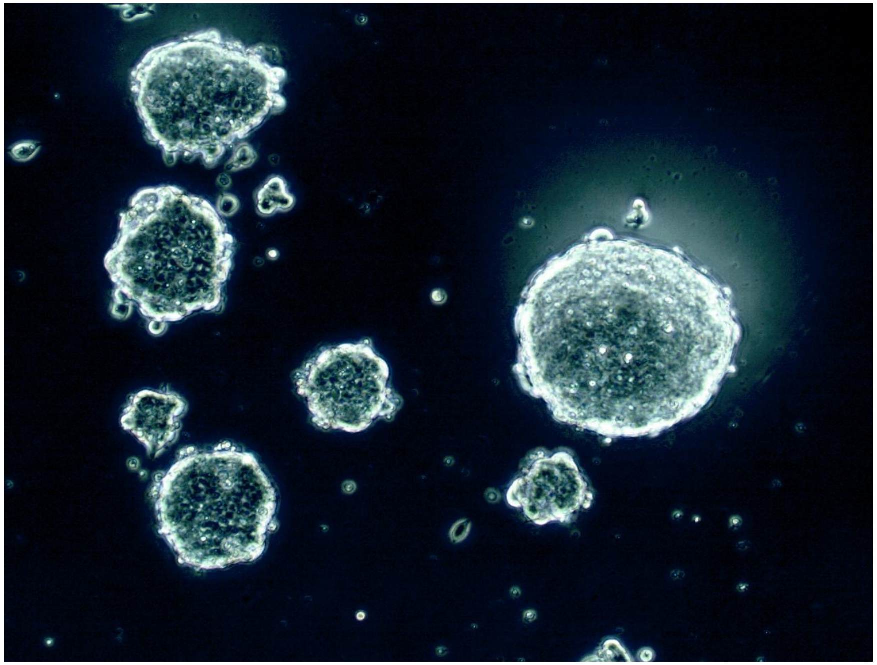

B.

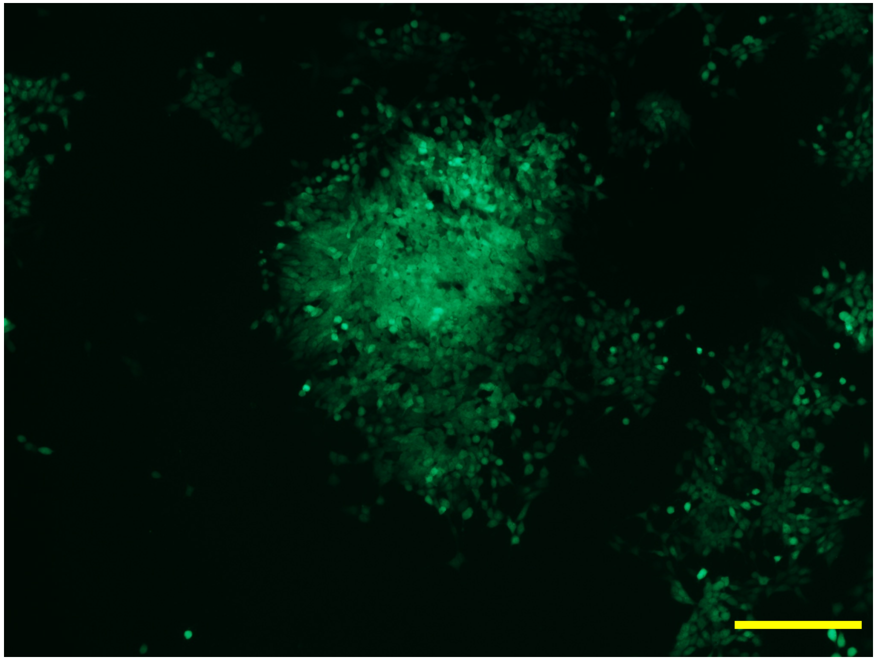

Supplementary Figure 2

A. Immunocytochemistry of pluripotent proteins on day 0 TNG-A mESCs

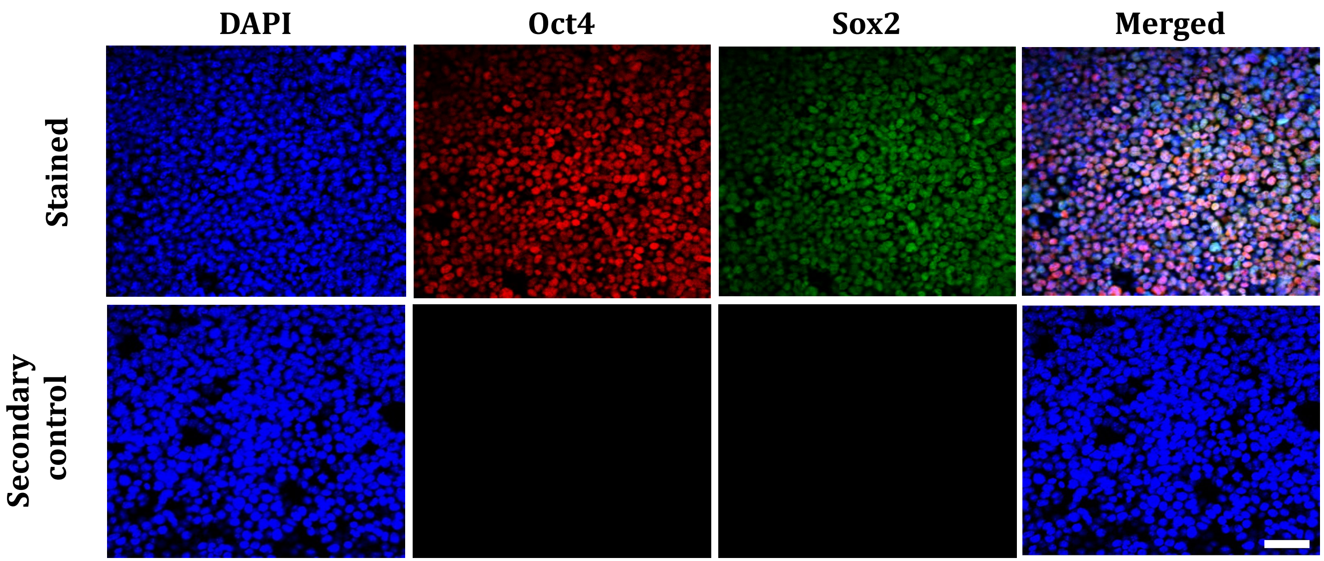

B. RT-PCR analysis on day 0

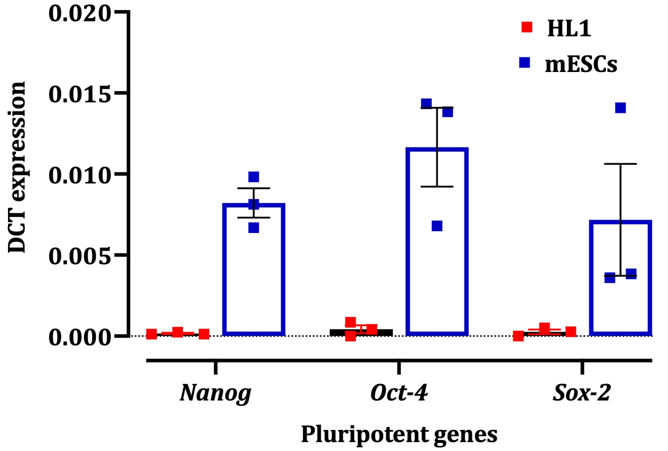

C. Immunocytochemistry of pluripotent proteins in E14 mESCs

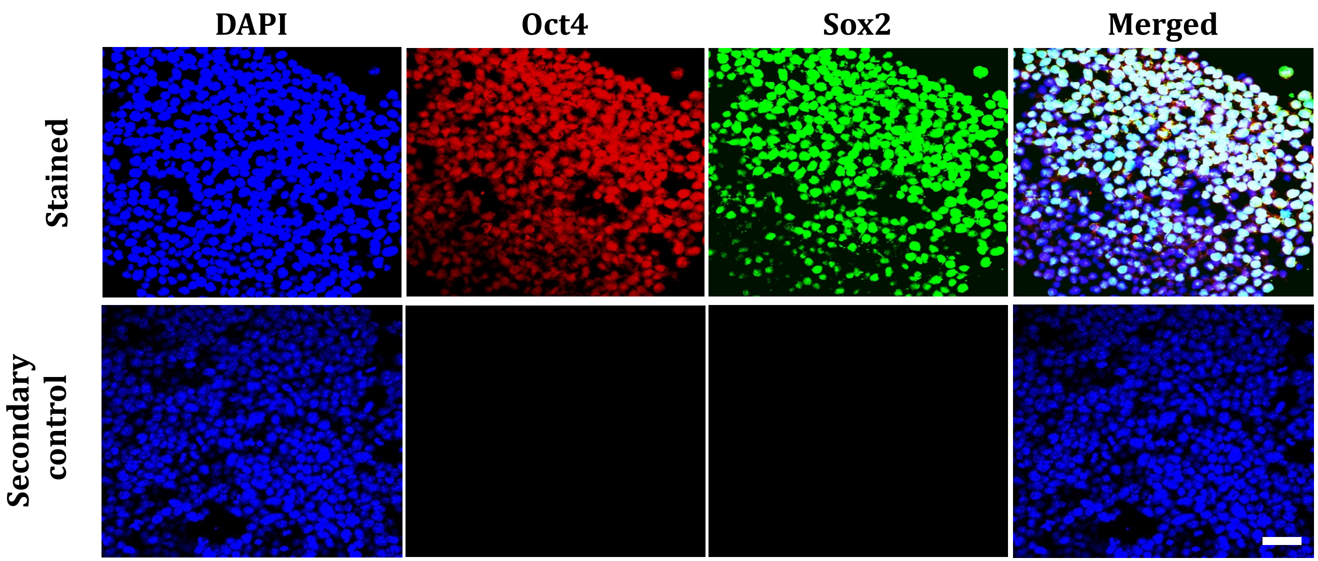

D. RT-PCR analysis on day 14

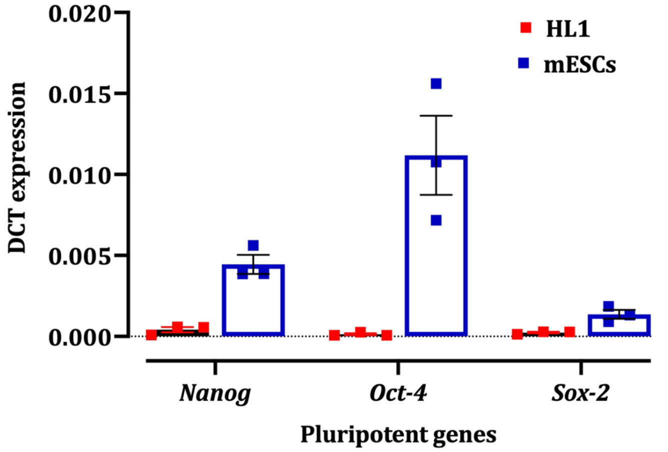

Supplementary Figure 3

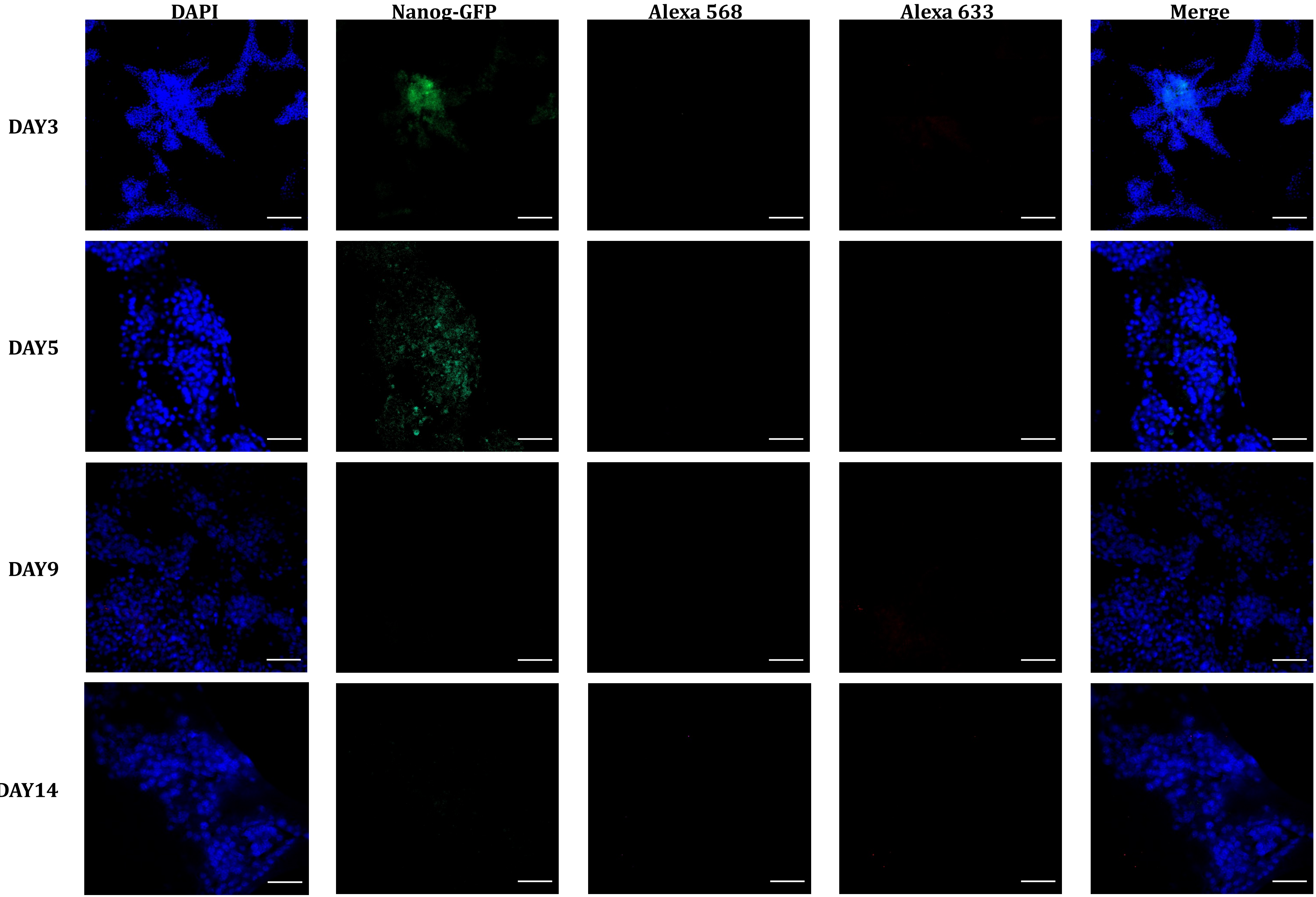

Supplementary Figure 4

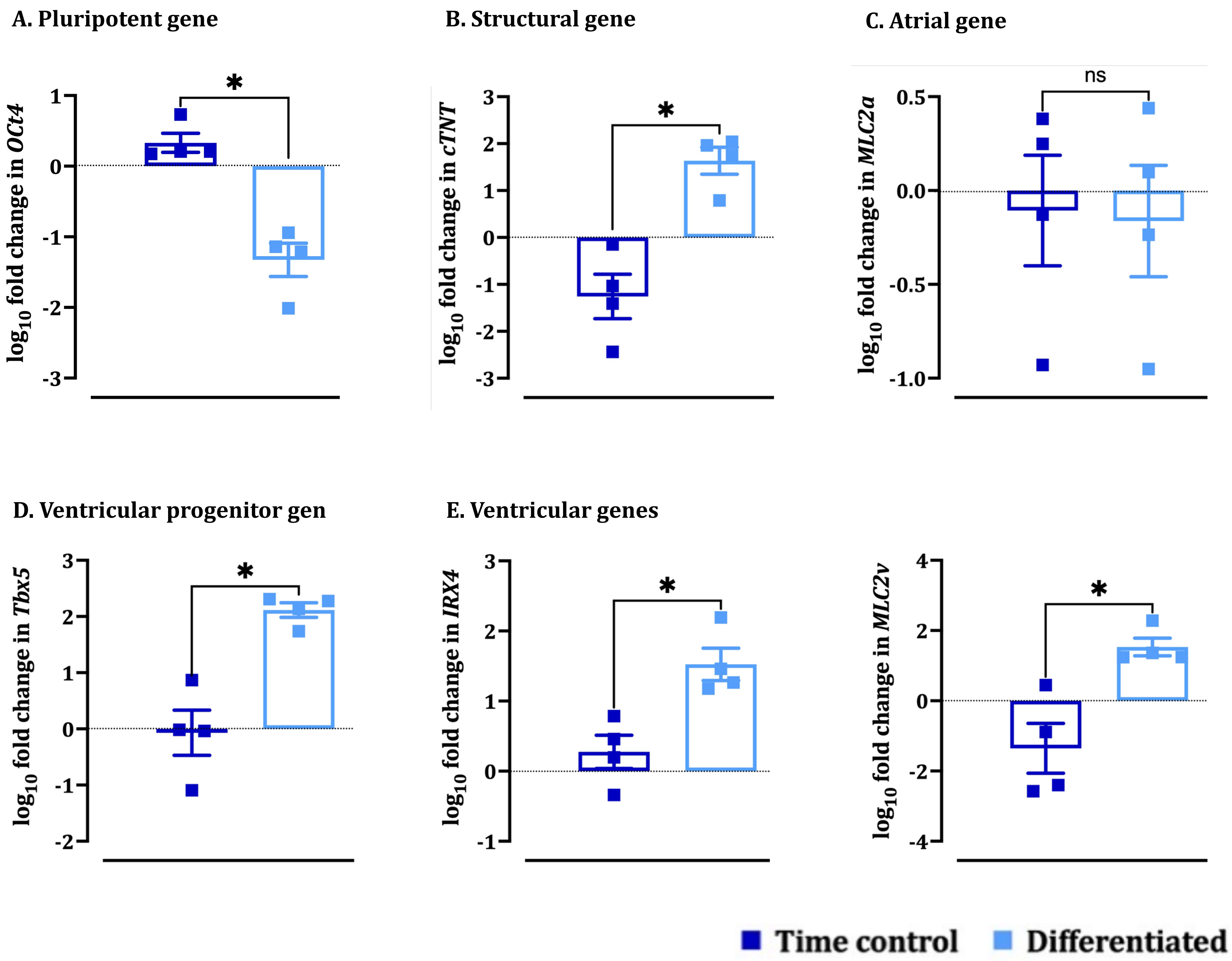

Supplementary Figure 5

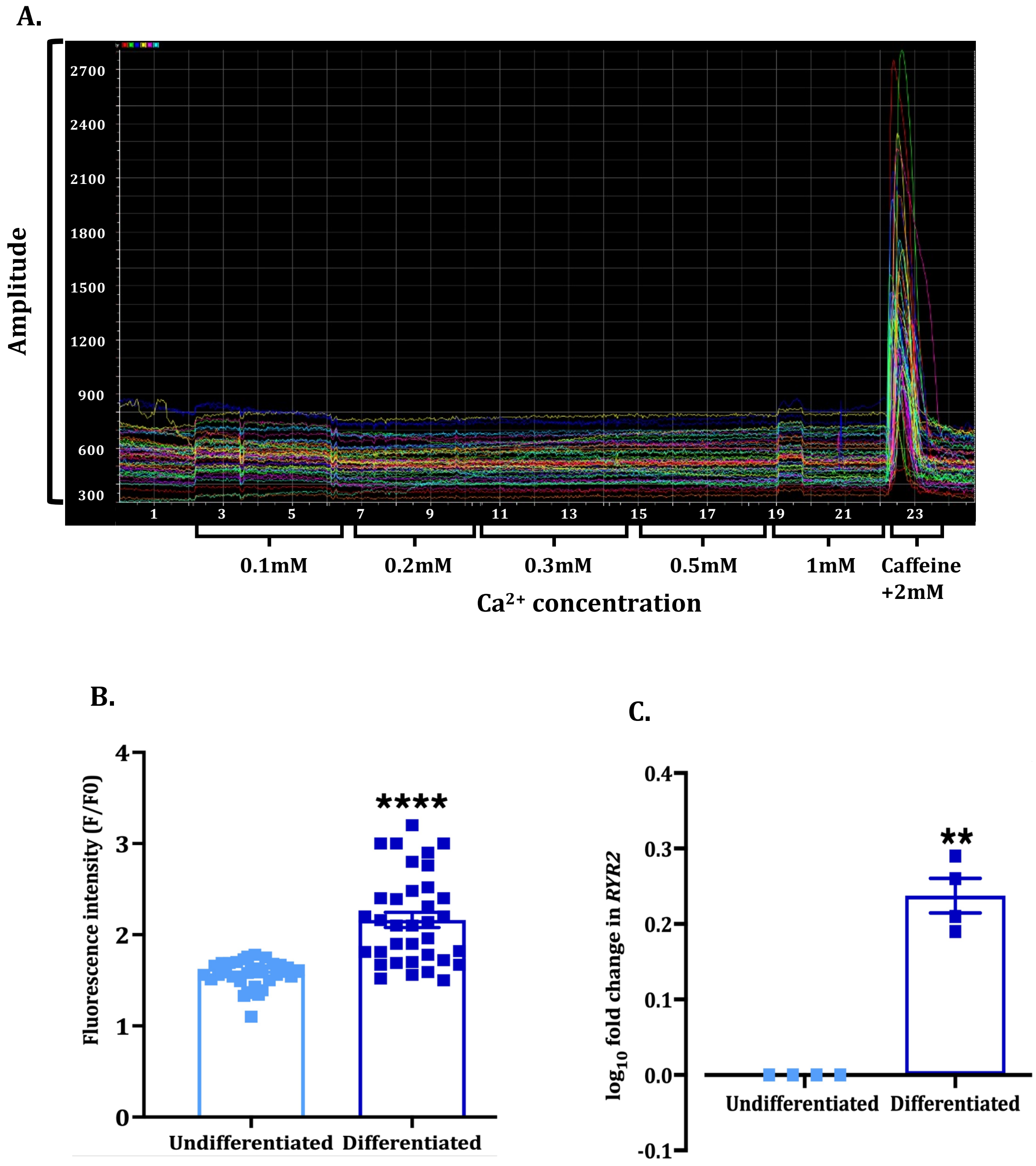

Supplementary Figure 6

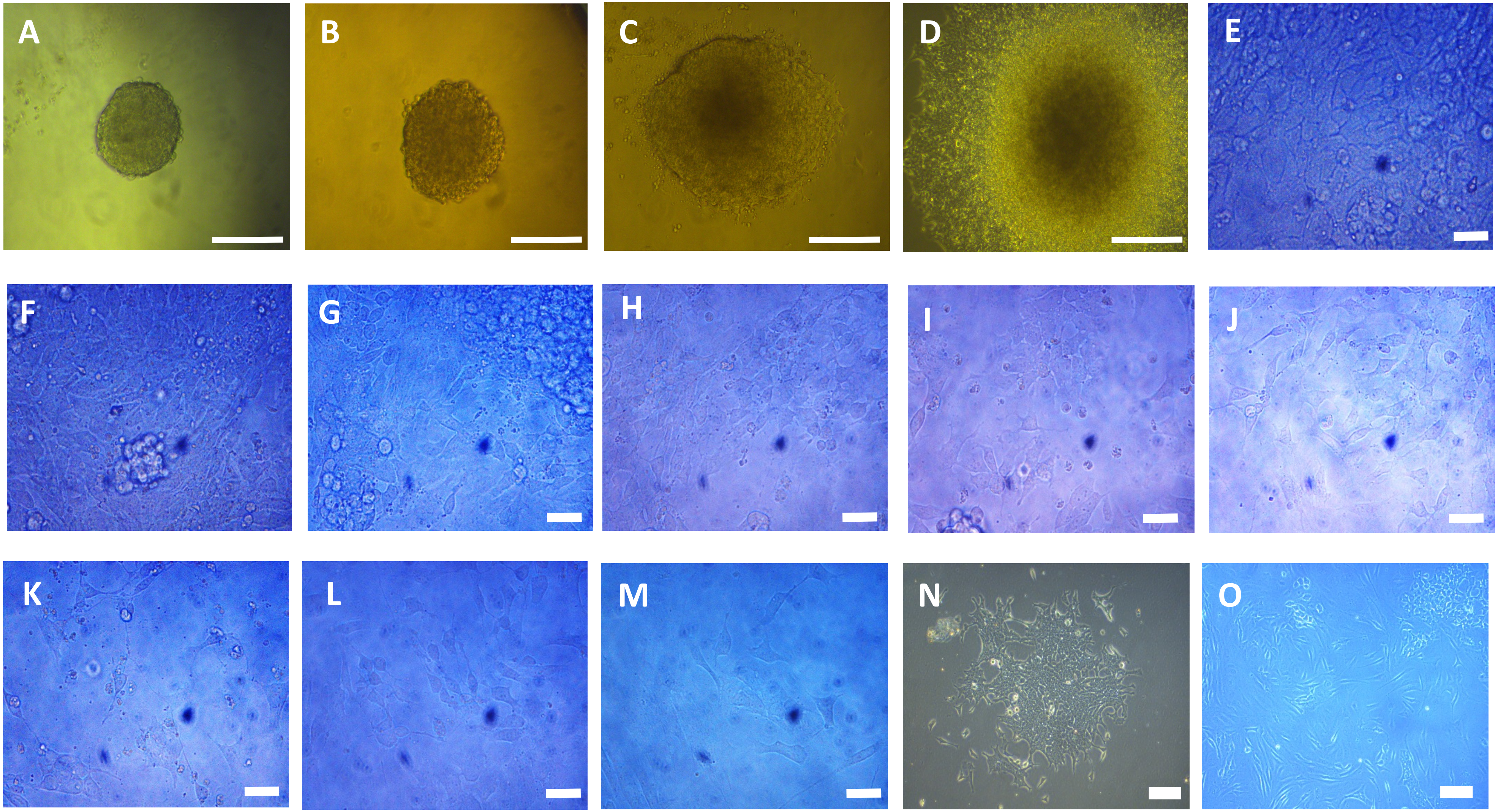

Supplemental Figure 7

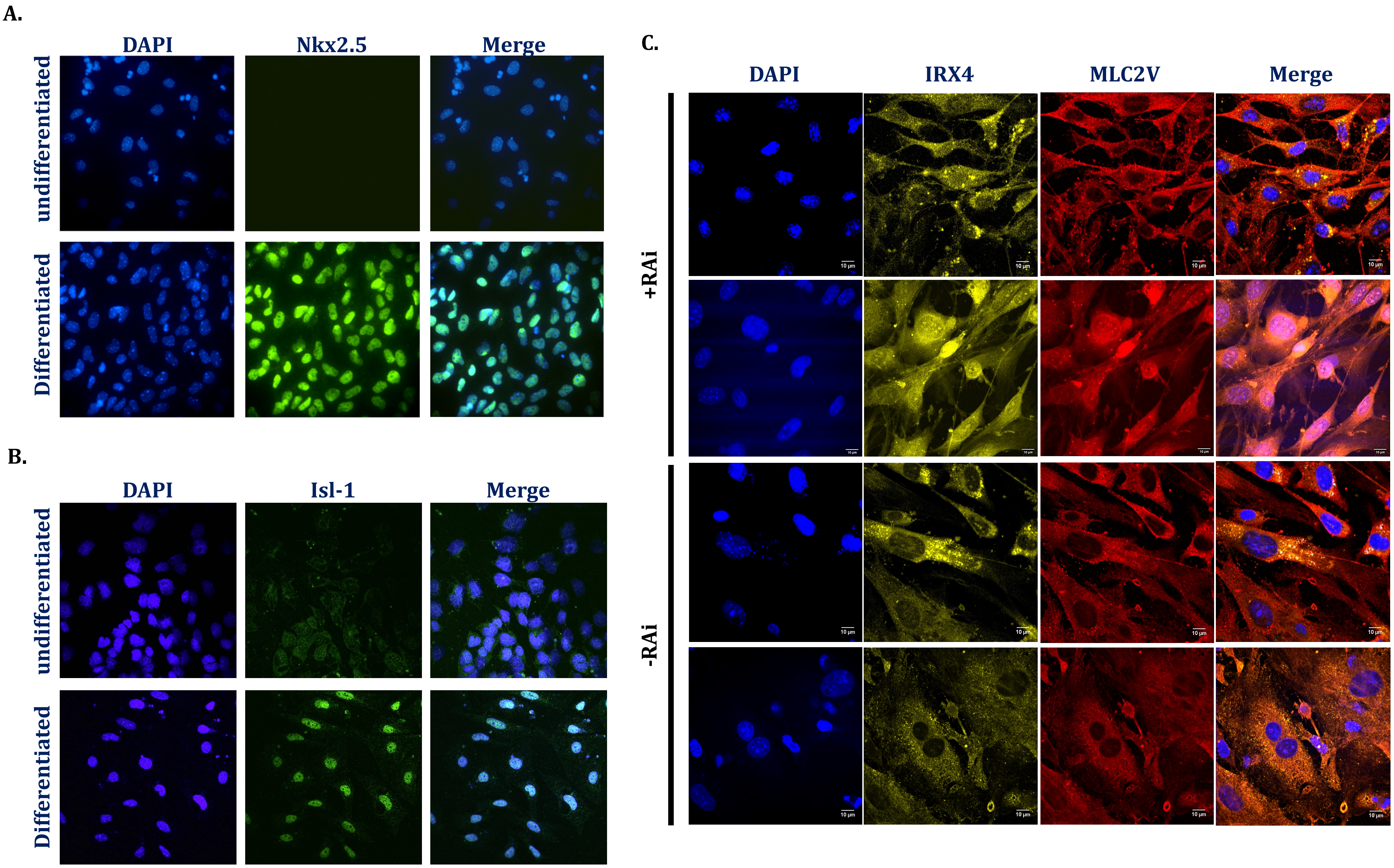

Supplemental Figure 7

D.

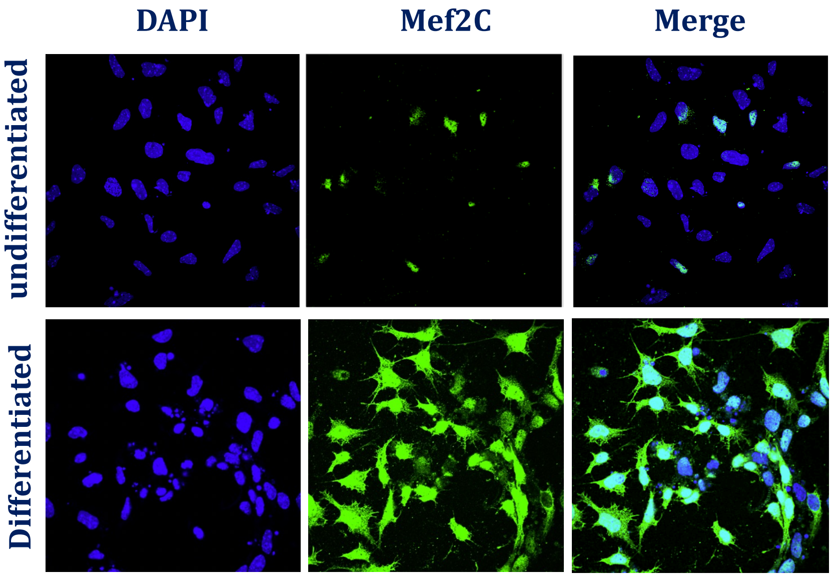

E.

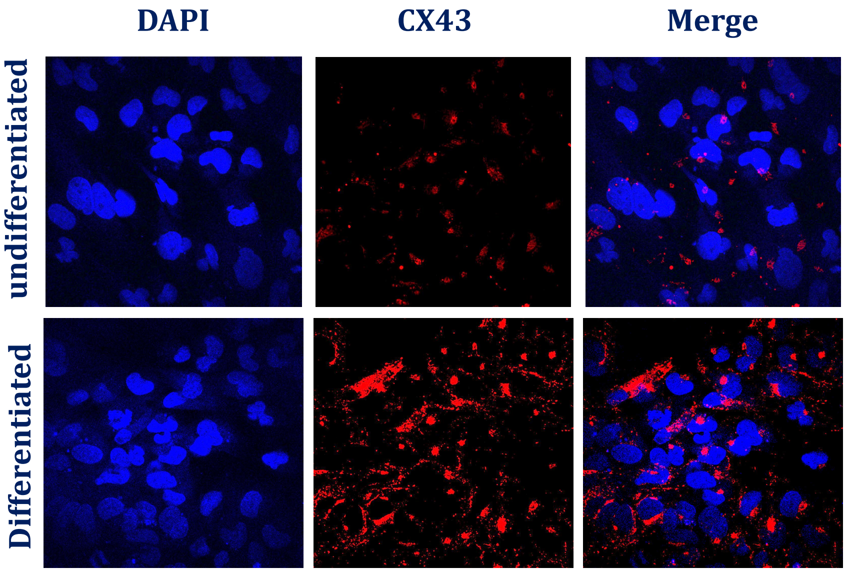

F.

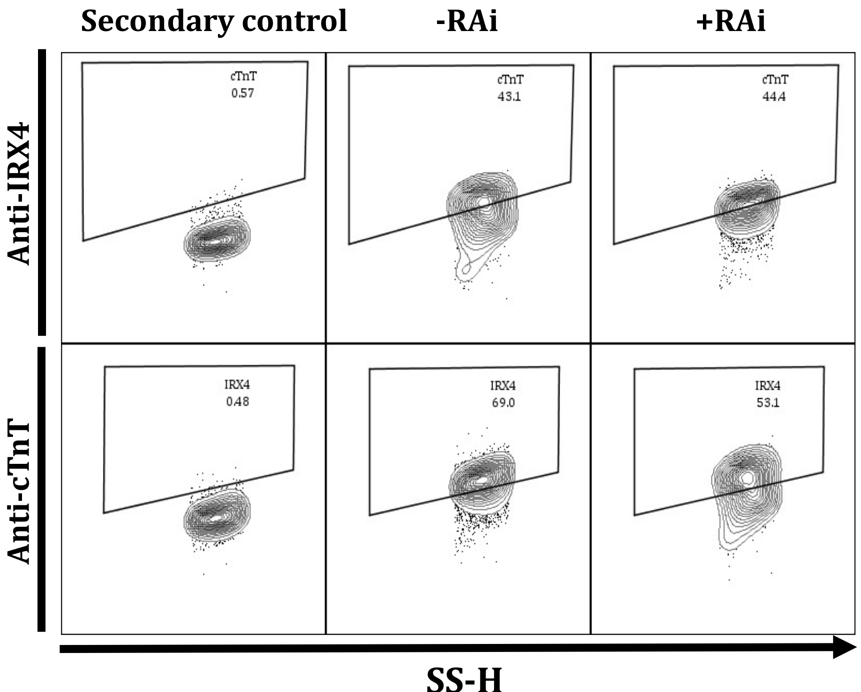

Supplemental Figure 8

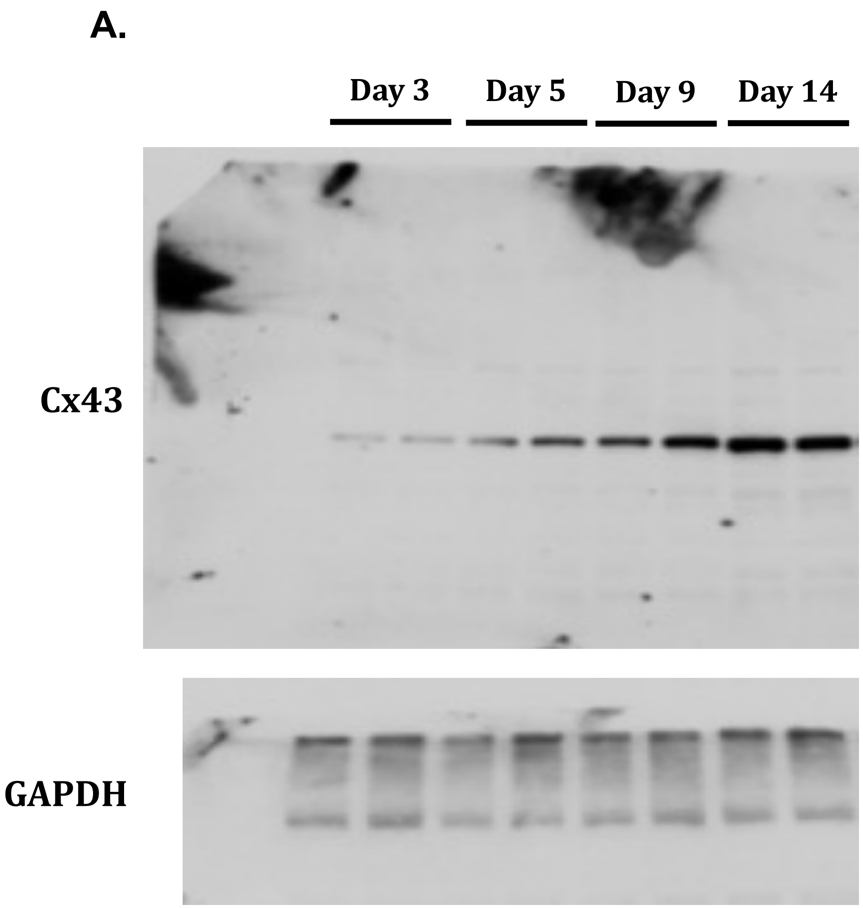

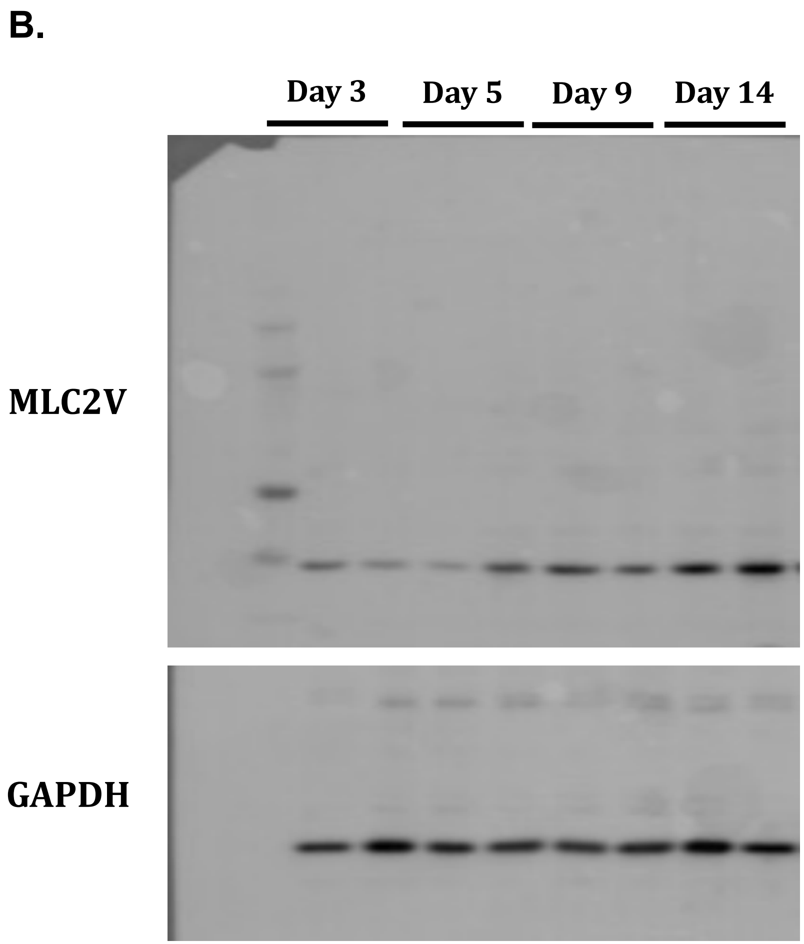

Supplement: Supplementary file 1 — Additional file 1. Figure S1. A. Representative bright-field image of TNG-A mESCs aggregated in spherical colonies. B. Acolony of TNG-A mESCs showing the endogenous expression of pluripotent nanog protein due to the GFP transgene tagged to nanog. Scale bar=100μm. Figure S2. A&C. Representative immunofluorescence images of mESCs on day 0 (A) and day 14 (time control, C) showing the expression of pluripotent markers (Oct4 and Sox2). Specificity of the antibody was confirmed by staining a independent samples with secondary controls only. B&D. Quantitative scater plot bar graphs of RT-PCr analysis showing the expression of pluripotent markers gene Nanog, Oct-4 and Sox-2 in of mESCs on day 0 (B) and day 14 (time control, G). Mature mouse cardiomyocyte cell line HL-1 cells were used as negative controls. Figure S3. Representative confocal microscopy images showing the cells at different days of differentiation that are stained with secondary antibodies with the same labelling procedure without the primary antibody treatment, to rule out the non-specific binding of the secondary antibody. Figure S4. Quantitative scatter plot bar graphs of RT-PCR analysis showing the expression of cardiac differentiation genes in mESCs grown in differentiation medium with treatments (differentiated) and mESCs grown without any treatment (time control). Data were analyzed by unpaired t-test presented as Mean ± SEM. *P<0.05. n=4 independent repeats. Figure S5. A. Representative image of Ca2+ activity in a single differentiated mESC-CM on day 14 (A). The cells did not show any propensity to spontaneously release the calcium when induced with different concentrations of Ca2+. B. Quantitative scatter plot bar graphs showing a significant response of differentiated cells to caffeine-induced Ca2+ release C. Quantitative scatter plot bar graphs of RT-PCR analysis showing the expression of RYR2 after 14 days of differentiation. Data were analyzed by unpaired t-test with Welch’s correction. **P<0.01 [file 13287_2022_2845_MOESM1_ESM.pdf]
